# Selective cellular localization of UHRF1 safeguards mammalian zygotic genome activation and early embryonic development

**DOI:** 10.1038/s41421-026-00896-3

**Published:** 2026-05-26

**Authors:** Rui Yan, Xin Cheng, Xin Long, Yating Zhu, Qiancheng Zhang, Fengyuan Sun, Fan Zhang, Mengyue Wang, Ruifeng Zhang, Tianzi Guo, Xinling Hou, Dongmei Ji, Yunxia Cao, Fei Gao, Dan Liang, Fan Guo

**Affiliations:** 1 State Key Laboratory of Organ Regeneration and Reconstruction, Institute of Zoology, Chinese Academy of Sciences, Beijing, China; 2 University of Chinese Academy of Sciences, Beijing, China; 3 Beijing Institute for Stem Cell and Regenerative Medicine, Beijing, China; 4 Department of Obstetrics and Gynecology, NHC Key Laboratory of Study on Abnormal Gametes and Reproductive Tract, The First Affiliated Hospital of Anhui Medical University, Hefei, Anhui China

**Keywords:** Epigenetics, Reprogramming

## Abstract

Zygotic genome activation (ZGA) is essential for initiating the developmental gene expression program in early embryos. However, whether a gating mechanism orchestrated by a limited number of factors exists in mammals remains debated. In this study, by utilizing an *Nlrp14*-deficient model that intriguingly disrupts the zygotic localization of UHRF1 and DNMT1, and in combination with comprehensive genetic approaches, we demonstrated that the nuclear exclusion of UHRF1 is essential for mouse ZGA and subsequent developmental progression. Mechanistically, the failure to exclude UHRF1 and DNMT1 from the nucleus in zygotes would impede DNA demethylation in LINE1 elements, promote UHRF1 binding to silence their expression, thereby reducing global chromatin accessibility and inhibiting ZGA. This effect was rescued in *Uhrf1/Nlrp14* double knockout (DKO) embryos, which still exhibited heavy DNA methylation, highlighting a dispensable role of UHRF1 in the maintenance of genome-wide DNA methylation after fertilization. Furthermore, reducing DNA methylation through *Dnmt1/Nlrp14* DKO or inhibiting the DNA methylation-binding domains of UHRF1 mitigated the adverse effects of nuclear-localized UHRF1 and reactivated the ZGA genes. Finally, we demonstrated that the residual nuclear UHRF1 in normal embryos binds to and facilitates the transcriptional inactivation of specific LTR subtypes that evade DNA demethylation during the genome-wide epigenetic reprogramming. Our findings not only highlight the biological significance of UHRF1 and DNMT1 nuclear exclusion but also elucidate the potentially conserved mechanism that regulates ZGA during mammalian preimplantation development.

## Introduction

Zygotic genome activation (ZGA) is critical for oocyte-to-embryo transition (OET) and is triggered by various maternal-effect genes (MEGs)^1^. In mice, ZGA predominantly occurs in 2-cell embryos and is initiated through interactions between transcription factors (TFs) and remodelled chromatin^2^. Additionally, previous studies have also demonstrated that RNA transcripts of transposable elements (TEs), such as LINE1, are essential for ZGA and preimplantation development^3–5^. Despite the extensive documentation of ZGA TFs in mammals^6–11^, the dynamic regulation and functional roles of TEs during ZGA within the context of chromatin remodelling remain undetermined^12^. Chromatin remodelling upon fertilization in mammals starts with the formation of two parental pronuclei and is orchestrated by epigenetic factors that function as histone-modifying enzymes or chromatin remodellers to facilitate chromatin accessibility and higher-order genome organization^13^. Notwithstanding the intimate link between chromatin remodelling and ZGA, the direction of causality is unclear^14^. The situation becomes more intricate when considering DNA methylation, which is extensively erased in preimplantation embryos and is correlated with both transcription and chromatin structure^15^.

The retention of DNMT1/UHRF1 mainly in the cytoplasm during early embryonic development is previously considered essential for passive DNA demethylation in the maternal genome^16,17^. Concurrently, the residual DNMT1/UHRF1 that remains in the nucleus is necessary to maintain DNA methylation at imprinted loci^18^. TET3-mediated 5mC oxidation mainly plays a role in paternal DNA demethylation, yet both ZGA and preimplantation development still occur in knockout embryos^19–21^. Therefore, the causal role of DNA methylation dynamics and its interplay with chromatin in early embryo development remains to be elucidated. The key for dissecting causal links between the major events that accompany ZGA is the choice of a genetic model that could intrinsically perturb these processes. In this study, we utilized our recently established NLRP14 deficiency model^22^, which disrupts the localization of DNMT1/UHRF1 and impedes DNA demethylation and ZGA. By integrating this model with comprehensive genetic approaches, we systematically investigated the causal relationships among chromatin remodelling, DNA demethylation, and transcriptional activation in early embryos. Therefore, we elucidated a mechanistic framework wherein UHRF1 regulates TEs activity through DNA methylation to ensure proper ZGA and preimplantation development in mammals.

## Results

### Spatiotemporal regulation of the subcellular localization of UHRF1 by NLRP14

**Fig. 1:**
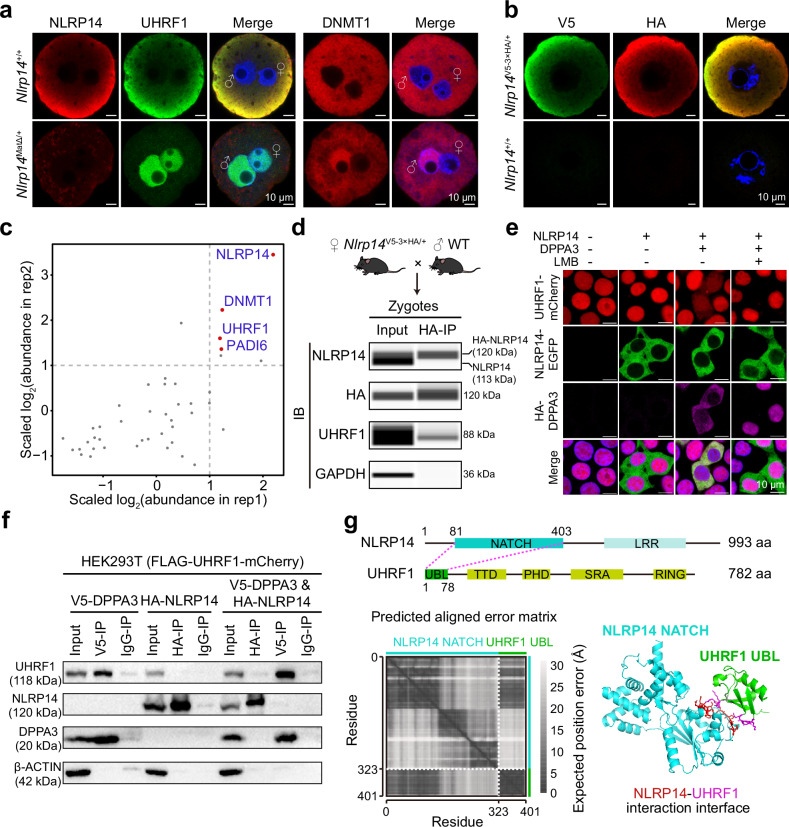
UHRF1 colocalized with and associated with NLRP14 in mouse zygotes. **a** Immunofluorescence staining of NLRP14, UHRF1, and DNMT1 in WT and *Nlrp14*^*mat-KO*^ mouse zygotes. Nuclei were counterstained with DAPI. For NLRP14 and UHRF1 co-staining: WT, *n* = 8; *Nlrp14*^*mat-KO*^, *n* = 9. For DNMT1 and UHRF1 co-staining: WT, *n* = 10; *Nlrp14*^*mat-KO*^, *n* = 16. Although DNMT1 and UHRF1 were co-stained, only DNMT1 is shown in **a**; quantification of UHRF1 signals is presented in Supplementary Fig. S1f. **b** Immunofluorescence staining of V5- and HA-tagged NLRP14 in *Nlrp14*^*V5-3×HA/+*^ knock-in (*n* = 8) and WT (*n* = 6) GV oocytes. **c** LC-MS/MS analysis of proteins co-immunoprecipitated with HA-NLRP14 in mouse *Nlrp14*^*V5-3×HA/+*^ zygotes from two independent experiments. UHRF1, DNMT1 and PADI6 (highlighted with a red dot) are among the top enrichment candidates. **d** Immunoblotting for NLRP14, HA-NLRP14, UHRF1 and GAPDH in mouse zygotes following anti-HA IP, performed via a capillary western blot (WES). **e** Immunofluorescence staining showing the subcellular localization of UHRF1 in HEK293T cells after ectopic expression of DPPA3 or NLRP14, with or without leptomycin B (LMB) treatment to inhibit nuclear export. **f** IP assay to examine protein interactions between UHRF1 and NLRP14 in HEK293T cells, with DPPA3 serving as a positive control for proteins known to interact with UHRF1. **g** The protein-protein interaction between mouse NLRP14 and UHRF1 was predicted using AlphaFold-3. Predicted aligned error (PAE) plots (left panel) showed that the top-scoring model of the NLRP14-UHRF1 interaction involves the NLRP14 NATCH domain and the UHRF1 UBL domain. The predicted structural model, with the highlighted interaction interface, was also shown (right panel).

We previously found that in the absence of the maternal factor NLRP14, zygotic DNMT1/UHRF1 translocate into the pronucleus and ZGA is severely inhibited, DNA demethylation is impaired, and embryos arrest at the 2-cell stage^22^. However, the detailed mechanism and interactions among these MEGs at the protein level have not been determined. We first generated an antibody against NLRP14 (Supplementary Fig. S1a), which successfully recognized this antigen in ectopically expressed HEK293T cells (Supplementary Fig. S1b, c). NLRP14 was colocalized with UHRF1 in oocytes, but knockout (KO) of this gene did not disturb the cytoplasmic location of either DNMT1 or UHRF1 (Supplementary Fig. S1d). However, the stability of DNMT1/UHRF1 was compromised in the absence of NLRP14 in oocytes (Supplementary Fig. S1e). Notably, only following fertilization, loss of maternal NLRP14 led to aberrant nuclear accumulation of DNMT1 in both the male and female pronuclei, with a more pronounced enrichment in the male pronucleus, and similarly led to significant changes in UHRF1 localization in both pronuclei (Fig. 1a; Supplementary Fig. S1f), suggesting that DNMT1/UHRF1 may be associated with NLRP14 in zygotes. A knock-in mouse model, in which V5 and hemagglutinin (HA) tags were tandemly linked to NLRP14, was utilized to further validate the interaction between UHRF1 and NLRP14 in zygotes through HA-antibody immunoprecipitation (IP), followed by mass spectrometry and immunoblot analysis (Fig. 1b–d). In addition to UHRF1, DNMT1 and PADI6 were confidently identified in the immunoprecipitation–mass spectrometry (IP-MS) analysis (Fig. 1c). Furthermore, nuclear accumulation of UHRF1 was observed in *Padi6*-deficient zygotes^23^, indicating that UHRF1, NLRP14, and PADI6 may assemble into a functional complex in mouse zygote. Interestingly, NLRP14 could not form homomeric complexes, as IP against HA-NLRP14 did not yield the wild-type (WT) NLRP14 protein (Fig. 1d). To determine whether the UHRF1-NLRP14 interaction involves the subcortical maternal complex (SCMC)^24^, we coexpressed NLRP14 and the SCMC core component NLRP5 in HEK293T cells (Supplementary Fig. S1g, h), and these two proteins did not show a strong interaction (Supplementary Fig. S1i). Moreover, KO of *Nlrp14* did not affect NLRP5 localization or SCMC protein expression in oocytes or early embryos (Supplementary Fig. S1j, k). Thus, zygotic UHRF1 interacted with NLRP14, which may be independent of the classic SCMC function.

Because DPPA3 (STELLA) physically interacts with UHRF1 during oogenesis^25^ and in somatic cell lines (Supplementary Fig. S2a), we subsequently examined the interactions among UHRF1, NLRP14 and DPPA3. The expression and distribution of DPPA3 in *Nlrp14* KO oocytes and zygotes were unaffected (Supplementary Figs. S1k, S2b). Unlike DPPA3, which was sufficient to dislodge UHRF1 into the cytoplasm by overexpression in HEK293T cells (Supplementary Fig. S2c), overexpression of NLRP14 alone had no such effect (Fig. 1e). Additionally, the overexpressed NLRP14 remained in the cytoplasm even in the presence of the exportin-1-specific inhibitor leptomycin B (LMB) (Fig. 1e), which was very different from DPPA3 that shuttled between the nucleus and cytoplasm (Fig. 1e; Supplementary Fig. S2c). We then co-expressed NLRP14 and DPPA3 in the stable UHRF1-mCherry HEK293T cell line to examine whether NLRP14 could interact with UHRF1 or DPPA3 in this context (Fig. 1e; Supplementary Fig. S2d). Despite the strong interaction between UHRF1 and DPPA3 (Fig. 1f), as previously reported^25^, NLRP14 exhibited no mutual effect on either protein (Fig. 1f), suggesting the interaction between UHRF1 and NLRP14 observed in zygotes was not conserved in somatic cells. Furthermore, UHRF1 was exported from the nucleus via a DPPA3-controlled mechanism rather than being retained in the cytoplasm by NLRP14 during oogenesis (Supplementary Fig. S2e–g). Nonetheless, UHRF1 was robustly maintained in the cytoplasm through the NLRP14-regulated mechanism after fertilization, while the DPPA3-linked export activity was largely attenuated owing to its nuclear localization (Supplementary Fig. S2h–k). Due to the inability to map the interaction between UHRF1 and NLRP14 in somatic cell lines, we employed AlphaFold-3 to predict their interaction domains. This approach yielded a high-confidence prediction of the UBL-NATCH interaction interface between UHRF1 and NLRP14 (Fig. 1g; Supplementary Table S1). These results suggest that during the oocyte-to-zygote transition, cytoplasmic retention of UHRF1 is primarily facilitated by NLRP14 rather than its nuclear export mediated by DPPA3.

### Nuclear invasion of UHRF1 coincides with defective chromatin remodelling in *Nlrp14*^*mat-KO*^ embryos

**Fig. 2:**
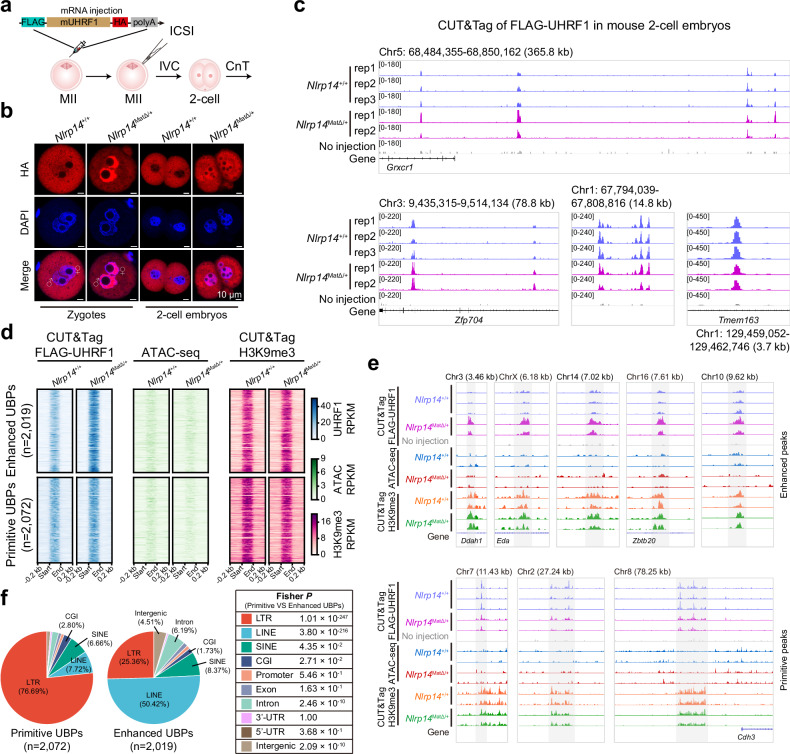
Nuclear inclusion of UHRF1 in *Nlrp14*^*mat-KO*^ embryos was linked to decreased chromatin accessibility. **a** Schematic illustration of the FLAG-UHRF1 CUT&Tag experimental approach in mouse 2-cell embryos. ICSI denotes intracytoplasmic sperm injection; IVC denotes in vitro culture; CnT denotes CUT&Tag. **b** HA staining was performed on WT and *Nlrp14*^*mat-KO*^ zygotes (WT, *n* = 7; KO, *n* = 14) and 2-cell embryos (WT, *n* = 11; KO, *n* = 16), following microinjection of FLAG-mUHRF1-HA mRNA. **c** Track plot showing the FLAG-UHRF1 binding signals in FLAG-UHRF1-HA mRNA microinjected WT and *Nlrp14*^*mat-KO*^ 2-cell embryos. CnT in WT 2-cell embryos without injection served as the negative control. **d** Heatmap showing the signals of FLAG-UHRF1, chromatin accessibility (ATAC), and H3K9me3 on enhanced and primitive UBPs in WT and *Nlrp14*^*mat-KO*^ 2-cell embryos. **e** Track plot showing the representative enhanced or primitive UBPs. The signals of chromatin accessibility (ATAC) and H3K9me3 in these regions in WT and *Nlrp14*^*mat-KO*^ 2-cell embryos were also shown. The rectangular shadow box represents the UBP regions. Chromosomal coordinate information for the regions shown in this figure includes: Chr3: 145,526,841-145,530,298 (3.46 kb), ChrX: 97,369,142-97,375,320 (6.18 kb), Chr14: 7,384,612-7,391,635 (7.02 kb), Chr16: 42,981,495-42,989,107 (7.61 kb), Chr10: 46,301,969-46,311,585 (9.62 kb); Chr7: 22,759,251-22,770,680 (11.43 kb), Chr2: 39,624,340-39,651,580 (27.24 kb), Chr8: 108,972,301-109,050,547 (78.25 kb). **f** Pie chart showing the genomic distribution of primitive or enhanced UBPs identified in (**d**). Statistical differences in the distributions between primitive and enhanced UBPs were assessed using Fisher’s exact test, with the corresponding *P* values summarized in the panel on the right.

To investigate the functional role of nuclear UHRF1 in early embryos, we conducted CUT&Tag assay of UHRF1 in both WT and *Nlrp14*^*mat-KO*^ 2-cell embryos. We evaluated several commercial UHRF1 antibodies in the stable FLAG-UHRF1-mCherry HEK293T cell line. Unfortunately, none of these antibodies could reliably detect UHRF1 signals in low-input cell numbers (500 cells), as effectively as the FLAG antibody (Supplementary Fig. S3a, b). Consequently, we employed the expression of FLAG-UHRF1-HA from the zygote stage onward and harvested embryos at the 2-cell stage for CUT&Tag analysis (Fig. 2a, b). The results showed reproducible and robust detection of UHRF1 signals in both WT and mat-KO embryos (Fig. 2c; Supplementary Fig. S3c and Table S2). We performed ATAC-seq using low input cells, validated in HEK293T cells (Supplementary Fig. S3d–f), to assess chromatin remodelling in 2-cell embryos. In combination with the comparison of our results with two previously published ATAC-seq datasets of mouse 2-cell embryos^26,27^, we demonstrated reliable detection of ATAC signals in our experiments (Supplementary Fig. S3g, h and Table S2). Both the number of ATAC peaks and chromatin accessibility around transcription start site (TSS) were significantly reduced in *Nlrp14*^*mat-KO*^ 2-cell embryos, as evidenced by the ATAC-seq results (Supplementary Fig. S3i–l). These findings suggest a correlation between aberrant UHRF1 localization and impaired chromatin remodelling in mat-KO embryos.

### UHRF1 binds to TEs and is associated with inaccessible chromatin

After stringent quality control, 3405 and 4999 UHRF1 binding peaks (UBPs) were robustly identified in the WT and *Nlrp14*^*mat-KO*^ 2-cell embryos among biological replicates (WT, *n* = 3; KO, *n* = 2), respectively (Supplementary Table S3). Among them, 2072 UBPs were primitively present in the WT embryos and were largely maintained in inaccessible states in both WT and *Nlrp14*^*mat-KO*^ embryos (Fig. 2d, e; Supplementary Table S4). Interestingly, 2019 UBPs were weakly detected in the WT embryos but presented significantly increased signal intensities in the *Nlrp14*^*mat-KO*^ embryos (Fig. 2d, e; Supplementary Table S4). Moreover, these enhanced UBPs in the *Nlrp14*^*mat-KO*^ embryos were likewise associated with closed chromatin regions (Fig. 2d, e). These results suggest that intensive physical binding of UHRF1 results in chromatin being maintained in inaccessible states. We next analysed the genomic features of these UBPs. The UBPs were not randomly distributed in the genome, but highly enriched at TEs such as LTRs and LINEs (Fig. 2f). Notably, compared to the primitive UBPs, the enhanced UBPs represented a substantial increase to LINE elements in *Nlrp14*^*mat-KO*^ 2-cell embryos (Fig. 2f).

### UHRF1 binding signals were associated with H3K9me3 in 2-cell embryos

How was UHRF1 deposited to chromatin in early embryos? Because UHRF1 recognizes H3K9me2 and H3K9me3 through its tandem Tudor domain (TTD) in somatic cells^28^, we performed immunostaining and CUT&Tag analysis of H3K9me3, the predominant heterochromatic mark in preimplantation embryos, in WT and *Nlrp14*^*mat-KO*^ embryos (Supplementary Fig. S4a–h). The overall signal intensity (Supplementary Fig. S4a–c) and distribution of H3K9me3 (Supplementary Fig. S4d–h) seemed undisturbed in the *Nlrp14*^*mat-KO*^ embryos. Indeed, obvious signals corresponding to UHRF1 binding were detected at H3K9me3 sites in the WT and mat-KO embryos (Supplementary Fig. S4f). Moreover, those enhanced and primitive UBPs were also exhibited H3K9me3 modifications (Fig. 2d, e). Therefore, the association between UHRF1 chromatin binding and H3K9me3 depositions in 2-cell embryos was observed. Interestingly, the enhanced UBPs identified in *Nlrp14*^*mat-KO*^ embryos also showed comparable H3K9me3 intensities in WT embryos (Fig. 2d, e). To quantitatively assess the biological relevance of these signals at UBPs, we performed profile analyses and evaluated the effect size. The result showed that only UHRF1 signals at enhanced UBPs exhibit a substantial effect size (Cohen’s d > 0.8) between WT and *Nlrp14*^*mat-KO*^ embryos (Supplementary Fig. S4i). In contrast, ATAC-seq and H3K9me3 signals show consistently limited effect sizes across regions (Supplementary Fig. S4i), indicating that these differences are negligible and unlikely to be biologically meaningful. This suggests that additional modification other than H3K9me3 in mat-KO embryos, possibly DNA methylation, may contribute to increased chromatin binding of UHRF1.

### ZGA and 2-cell arrest can be rescued in *Nlrp14* and *Uhrf1* double maternal-KO embryos

NLRP14 is an MEG, and its deficiency may result in maternal degradation of undefined proteins that cause defective ZGA independent of UHRF1 function. To rule out this possibility, we generated (*Uhrf1*^*f/f*^, *Nlrp14*^*–/–*^, *Gdf9-Cre*) female mice deficient in both UHRF1 and NLRP14 in oocytes (*Uhrf1&Nlrp14*^*mat-DKO*^). Despite the decrease in efficiency to ~40%, *Uhrf1*^*mat-KO*^ embryos could develop into blastocysts (Supplementary Fig. S5a, c, e, f), as previously reported^25^. Since *Nlrp14* KO males are fully fertile^22^, we obtained (*Uhrf1*^*f/f*^, *Nlrp14*^*–/–*^, *Gdf9-Cre*) male mice and crossed them with (*Uhrf1*^*f/f*^, *Nlrp14*^*+/–*^) females to produce double mutant females with optimized efficiency (Supplementary Fig. S6a, b). The (*Uhrf1*^*f/f*^, *Nlrp14*^*–/–*^, *Gdf9-Cre*) mice were indistinguishable in terms of gonad morphology and gametogenesis compared to the WT mice (Supplementary Fig. S6c, d), and the male mice were still fully fertile (Supplementary Fig. S6e).

**Fig. 3:**
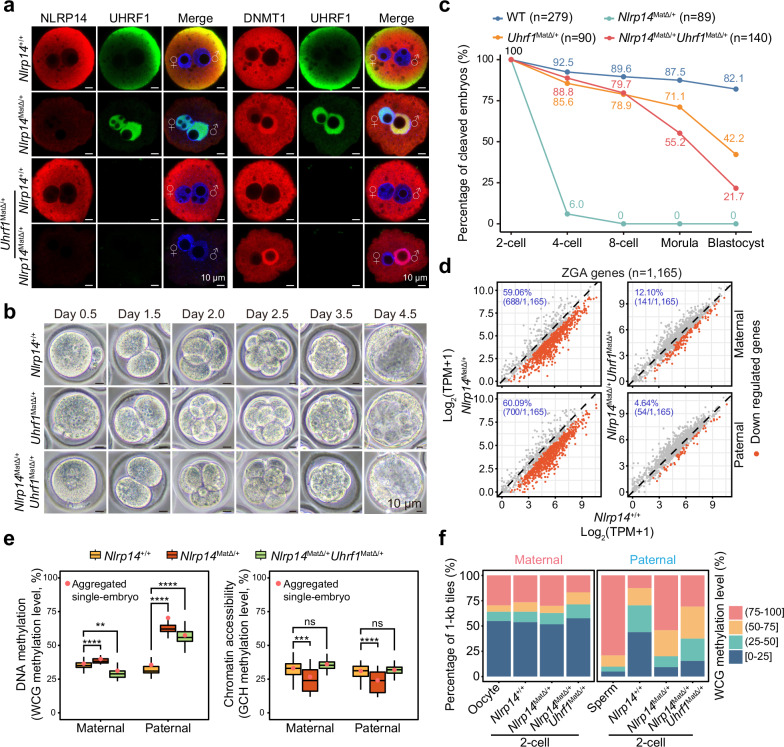
Maternal deficiency of UHRF1 in *Nlrp14*^*mat-KO*^ embryos rescued ZGA and unblocked 2-cell arrest. **a** Immunofluorescence staining of NLRP14, UHRF1 and DNMT1 in WT, *Nlrp14*^*mat-KO*^, *Uhrf1*^*mat-KO*^ and *Uhrf1&Nlrp14*^*mat-DKO*^ mouse zygotes. For NLRP14 and UHRF1 co-staining: WT, *n* = 15; *Nlrp14*^*mat-KO*^, *n* = 16; *Uhrf1*^*mat-KO*^, *n* = 15; *Uhrf1&Nlrp14*^*mat-DKO*^, *n* = 12. For DNMT1 and UHRF1 co-staining: WT, *n* = 14; *Nlrp14*^*mat-KO*^, *n* = 16; *Uhrf1*^*mat-KO*^, *n* = 7; *Uhrf1&Nlrp14*^*mat-DKO*^, *n* = 11. **b** Representative images of cleavage embryo morphology from WT, *Uhrf1*^*mat-KO*^ and *Uhrf1&Nlrp14*^*mat-DKO*^ mouse embryos. **c** Line plot showing the percentages of cleaved embryos at different stages. Embryos for photography were collected at 42–43 h (2-cell), 62–63 h (4-cell), 71–72 h (8-cell), 94–96 h (morula) and 120–121 h (blastocyst) after hCG injection. **d** Scatter plots showing changes in ZGA gene expression in *Nlrp14*^*mat-KO*^ and *Uhrf1&Nlrp14*^*mat-DKO*^ 2-cell embryos compared with WT embryos. Downregulated genes are shown as red dots. **e** Box plots showing the DNA methylation levels and chromatin accessibility in 2-cell embryos from the WT, *Nlrp14*^*mat-KO*^ and *Uhrf1&Nlrp14*^*mat-DKO*^ populations. “Aggregated single-embryo” refers to WCG/GCH methylation levels calculated from aggregated single-embryo data and is shown as red dots. Statistical significance was assessed using a two-tailed Student’s *t*-test. **f** Stacked bar plots showing the percentages of 1-kb tiles with categorical DNA methylation levels in MII oocytes, sperm, and 2-cell embryos with the indicated genotypes. DNA methylation levels are color-coded.

DNMT1 was detected mainly in the cytoplasm of WT or *Uhrf1*^*mat-KO*^ zygotes, but its signal could be observed in the nucleus of *Nlrp14*^*mat-KO*^ or *Uhrf1&Nlrp14*^*mat-DKO*^ zygotes (Fig. 3a). Interestingly, complete release of *Uhrf1&Nlrp14*^*mat-DKO*^ embryos occurred after 2-cell arrest, and 21.7% of these embryos developed to the blastocyst stage (Fig. 3b, c; Supplementary Fig. S6f, g). Thus, we conducted scChaRM-seq^29,30^ of individual 2-cell embryos to simultaneously evaluate changes at the molecular level, including gene expression, DNA methylation and chromatin accessibility. We first examined ZGA gene^8^ expression in the *Uhrf1&Nlrp14*^*mat-DKO*^ 2-cell embryos. A total of 59.06% and 60.09% of the ZGA genes were significantly downregulated (log_2_FC < –0.5 and FDR-adjusted *P* value < 0.05) in the maternal and paternal genomes of *Nlrp14*^*mat-KO*^ 2-cell embryos, respectively, but their expression levels were almost recovered in the *Uhrf1&Nlrp14*^*mat-DKO*^ embryos (Fig. 3d; Supplementary Fig. S7a and Table S5), except for very few genes (Fig. 3d). These results suggest that the nuclear invasion of UHRF1 is the main cause of ZGA blockade and 2-cell arrest in *Nlrp14*^*mat-KO*^ embryos.

### Chromatin accessibility is restored in *Uhrf1&Nlrp14*^*mat-DKO*^ embryos, but DNA demethylation is still globally hampered

Next, we investigated chromatin changes in *Uhrf1&Nlrp14*^*mat-DKO*^ 2-cell embryos. Surprisingly, the DNA demethylation of the paternal genome of individual *Uhrf1&Nlrp14*^*mat-DKO*^ 2-cell embryos was still impeded (Fig. 3e). For the maternal genome, global DNA methylation levels showed moderate changes that deviated from the passive demethylation rate between the individual WT, *Nlrp14*^*mat-KO*^, and *Uhrf1&Nlrp14*^*mat-DKO*^ 2-cell embryos (Fig. 3e). However, global chromatin accessibility was successfully recovered in both parental genomes of individual *Uhrf1&Nlrp14*^*mat-DKO*^ 2-cell embryos (Fig. 3e). We then aggregated the single-embryo sequencing data to increase genomic coverage. The global DNA methylation level and genomic distribution, especially those of the paternal genome, were nearly identical between the *Nlrp14*^*mat-KO*^ and *Uhrf1&Nlrp14*^*mat-DKO*^ embryos (Fig. 3f; Supplementary Fig. S7b, c). Germline imprinting control regions (gICRs), which maintain DNA methylation in preimplantation embryos, could maintain DNA methylation in the absence of UHRF1 in DKO embryos (Supplementary Fig. S7d). Moreover, the paternal loci that were demethylated upon fertilization were still hypermethylated in the *Uhrf1&Nlrp14*^*mat-DKO*^ embryos (Supplementary Fig. S7e and Table S6). However, chromatin accessibility was restored in the *Uhrf1&Nlrp14*^*mat-DKO*^ embryos, as indicated by the GCH methylation level at the genome-wide region, transcription start site (TSS), and CTCF-binding region (Fig. 3e; Supplementary Fig. S7c, f, g). Thus, these results, combined with the genomic distribution features of UHRF1 (Fig. 2), reveal an unusual role of excessive nucleus UHRF1 in early embryogenesis, negative regulation of chromatin accessibility but not participation in whole-genome DNA methylation maintenance.

### DNA methylation contributes to the detrimental role of excessive nuclear UHRF1 in early embryos

**Fig. 4:**
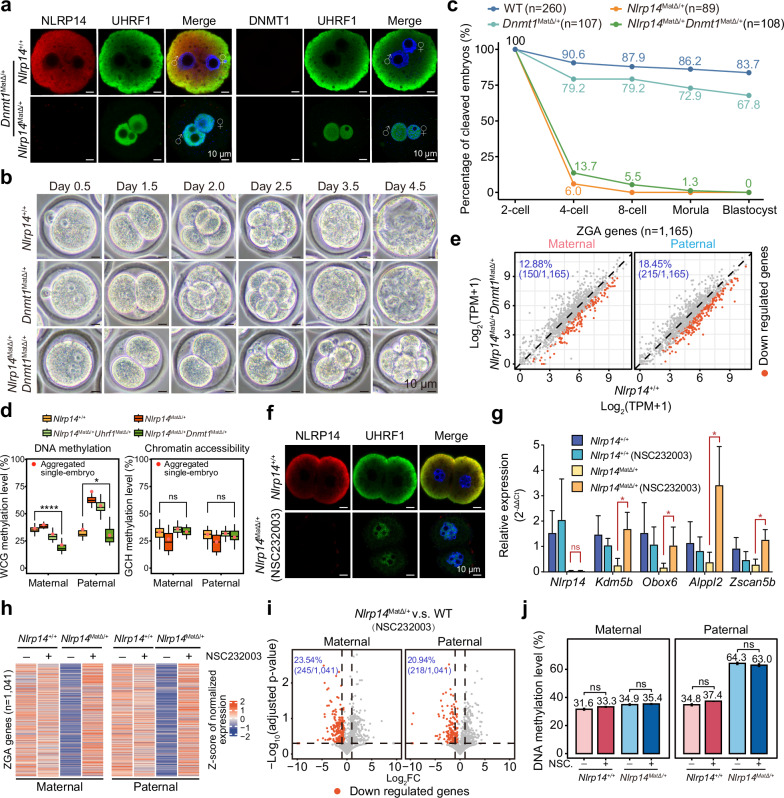
The chromatin occupancy of UHRF1 was modulated by DNA methylation and affected ZGA activation. **a** Immunofluorescence staining of NLRP14, UHRF1 and DNMT1 in *Dnmt1*^*mat-KO*^ and *Dnmt1&Nlrp14*^*mat-DKO*^ zygotes. For NLRP14 and UHRF1 co-staining: *Dnmt1*^*mat-KO*^, *n* = 3; *Dnmt1&Nlrp14*^*mat-DKO*^, *n* = 4. For DNMT1 and UHRF1 co-staining: *Dnmt1*^*mat-KO*^, *n* = 3; *Dnmt1&Nlrp14*^*mat-DKO*^, *n* = 5. **b** Representative images of cleaved embryo morphology from WT, *Dnmt1*^*mat-KO*^ or *Dnmt1&Nlrp14*^*mat-DKO*^ mouse embryos. **c** Line plot showing the percentages of cleaved embryos at different stages. **d** Box plots showing DNA methylation levels and chromatin accessibility in 2-cell embryos from the WT, *Nlrp14*^*mat-KO*^, *Uhrf1&Nlrp14*^*mat-DKO*^ and *Dnmt1&Nlrp14*^*mat-DKO*^ populations. “Aggregated single-embryo” refers to WCG/GCH methylation levels calculated from aggregated single-embryo data and is shown as red dots. Statistical significance was assessed using a two-tailed Student’s *t*-test. **e** Scatter plots showing changes in ZGA gene expression in *Dnmt1&Nlrp14*^*mat-DKO*^ 2-cell embryos compared with WT controls. Downregulated genes are shown as red dots. **f** Immunofluorescence staining of NLRP14 and UHRF1 in WT (*n* = 4) and NSC232003-treated *Nlrp14*^*mat-KO*^ 2-cell embryos (*n* = 5). **g** Bar plot showing the relative expression levels of representative ZGA genes in WT and *Nlrp14*^*mat-KO*^ 2-cell embryos with or without NSC232003 treatment. Each group includes 4 independent biological replicates. The error bars represent the mean ± SD. Statistical significance was assessed using Mann-Whitney. **h** Heatmap showing *Z*-scores of the normalized expression levels of ZGA genes in WT and *Nlrp14*^*mat-KO*^ 2-cell embryos with or without NSC232003 treatment. **i** Scatter plots showing changes in ZGA gene expression between WT and *Nlrp14*^*mat-KO*^ 2-cell embryos treated with NSC232003. Downregulated genes are shown as red dots. **j** Bar plots showing global DNA methylation levels of WT and *Nlrp14*^*mat-KO*^ 2-cell embryos with or without NSC232003 treatment. Significance was also calculated (Wilcoxon test). ns denotes not significant.

Although the above results demonstrated that global DNA demethylation is not needed for ZGA recovery in *Uhrf1&Nlrp14*^*mat-DKO*^ embryos, this process may regulate the interaction between UHRF1 and chromatin. Because UHRF1 can bind to chromatin through its SET- and RING-associated domain (SRA domain), which recognizes DNA methylation^28^. To test this possibility, we generated (*Dnmt1*^*f/f*^, *Nlrp14*^*–/–*^, *Gdf9-Cre*) female mice deficient in both DNMT1 and NLRP14 in oocytes (*Dnmt1&Nlrp14*^*mat-DKO*^) by using the similar strategy to that used for the (*Uhrf1*^*f/f*^, *Nlrp14*^*–/–*^, *Gdf9-Cre*) females (Supplementary Fig. S6). The *Dnmt1*^*mat-KO*^ embryos exhibited no obvious defects in preimplantation development (Supplementary Fig. S5b, d–f), as previously reported^18^. UHRF1 was still located in the nucleus in DKO zygotes (Fig. 4a). Interestingly, the *Dnmt1&Nlrp14*^*mat-DKO*^ embryos still exhibited severe 2-cell arrest (Fig. 4b, c; Supplementary Fig. S6f), suggesting that UHRF1 function could not be completely eradicated through the depletion of DNMT1. However, both the maternal and paternal genomes of individual *Dnmt1&Nlrp14*^*mat-DKO*^ embryos exhibited restored passive DNA demethylation at the genome-wide level (Fig. 4d; Supplementary Fig. S8a) and at different genomic elements (Supplementary Fig. S8b, c).

Despite the persistent nuclear localization of UHRF1 in *Dnmt1&Nlrp14*^*mat-DKO*^ embryos (Fig. 4a), chromatin accessibility was increased (Fig. 4d; Supplementary Fig. S8a), and the majority of ZGA genes were reactivated (Fig. 4e). However, 12.88% of maternal and 18.45% of paternal ZGA genes remained unrecovered in *Dnmt1&Nlrp14*^*mat-DKO*^ embryos (Fig. 4e; Supplementary Table S5). Additionally, mat-gICRs reduced their DNA methylation but maintained chromatin accessibility in *Dnmt1&Nlrp14*^*mat-DKO*^ embryos (Supplementary Fig. S8d). Notably, despite the depletion of UHRF1, the UBPs were found to be hypermethylated in *Uhrf1&Nlrp14*^*mat-DKO*^ embryos. In contrast, the UBPs were heavily demethylated in *Dnmt1&Nlrp14*^*mat-DKO*^ embryos (Supplementary Fig. S8e). Thus, DNA methylation may play a role in mediating chromatin binding by UHRF1, and the extent to which DNA methylation is decreased would partially eliminate the unfavourable effects of aberrant nuclear invasion of UHRF1 in early embryos.

### Inhibiting chromatin binding of UHRF1 through the SRA domain reestablishes ZGA genes in *Nlrp14*^*mat-KO*^ embryos

To further validate the relationship between chromatin binding of UHRF1 and hyper DNA methylation, we introduced a chemical inhibitor to specifically disrupt the chromatin binding of UHRF1 in *Nlrp14*^*mat-KO*^ embryos, which maintained the nuclear signals of DNMT1/UHRF1 (Fig. 4f). Mechanistically, NSC232003 binds to the 5mC binding pocket of the SRA domain^31^ to inhibit UHRF1 function. We first tested the expression of several known ZGA genes, such as *Kdm5b*, *Obox6*, *Alppl2* and *Zscan5b*, after treatment with NSC232003 (Fig. 4g). Interestingly, all of these genes were reactivated in the *Nlrp14*^*mat-KO*^ 2-cell embryos in the presence of NSC232003 (Fig. 4g). Moreover, 76.5% and 79.1% of the ZGA genes were re-expressed in the maternal and paternal genomes, respectively, of the NSC232003-treated *Nlrp14*^*mat-KO*^ 2-cell embryos (Fig. 4h, i; Supplementary Table S7). The global DNA methylation level, as assessed by whole-genome bisulfite sequencing, remained unaltered in both WT and *Nlrp14*^*mat-KO*^ 2-cell embryos following treatment with NSC232003 (Fig. 4j). These results further suggested that the excessive binding of UHRF1 via hyper DNA methylation may be the primary cause for the ZGA failure in *Nlrp14*^*mat-KO*^ embryos.

### Primitive UHRF1 binding in early embryos is necessary for LTR subclasses inactivation

We have uncovered the deleterious role of aberrant UHRF1 nuclear localization on ZGA failure in early embryos, prompting an intriguing question regarding the role of primitive UHRF1 binding in WT embryos. To address this, we continued to use the maternal *Uhrf1* knockout mouse model, in which depletion of full-length UHRF1 protein was confirmed (Supplementary Figs. S5, S9a, b). First, we assessed the expression of major ZGA genes in *Uhrf1*^*mat-KO*^ late zygotes and 2-cell embryos (Supplementary Fig. S9c, d). The obtained results validated that, regardless of UHRF1 presence, the major ZGA genes remained repressed in the late zygotic stage, while their expression was largely elevated in the late 2-cell stage (Supplementary Fig. S9c, d). These observations suggested that the primitive UHRF1 binding in WT embryos did not play a role in either blocking or promoting ZGA.

**Fig. 5:**
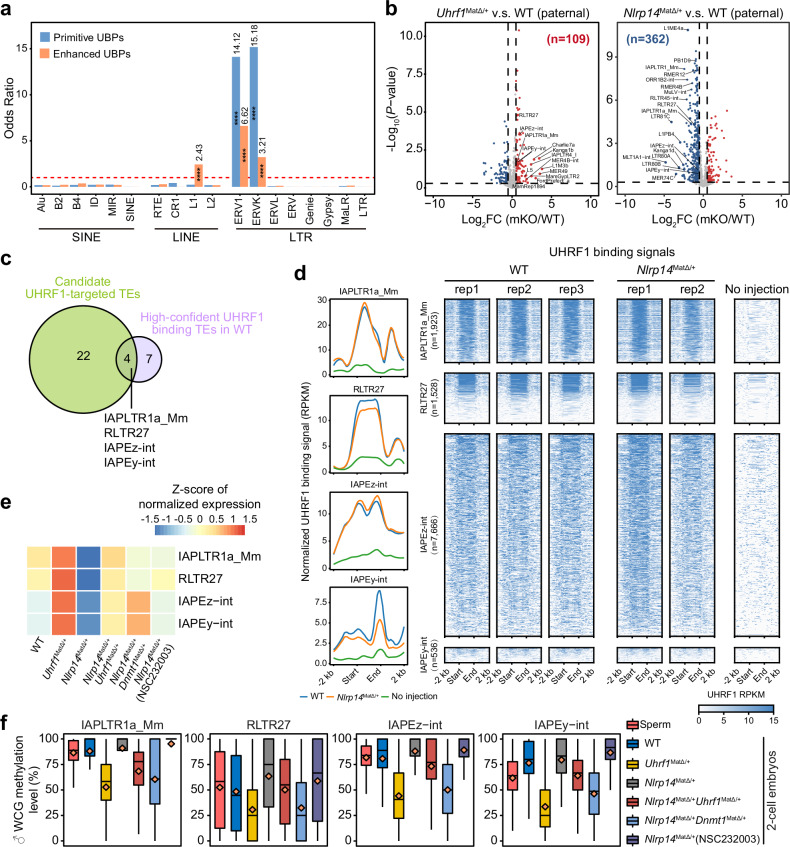
Primitive UHRF1 binding in early embryos maintains specific LTR subtypes in an inactive state. **a** Bar plot showing the enrichment of primitive and enhanced UBPs across TE subclasses, assessed by a permutation test with 10,000 random region sets matched to the real UBPs in both number and length. The dashed red line indicates an odds ratio of 1, corresponding to no enrichment relative to randomized background. Significantly enriched TE subclasses are labeled with their corresponding odds ratios and statistical significance compared with the randomized background (permutation-based empirical *P* values; ****P < 1 × 10^–^⁴). **b** Scatter plots showing changes in paternal transposable elements (TEs) expression in *Uhrf1*^*mat-KO*^ and *Nlrp14*^*mat-KO*^ 2-cell embryos compared with WT 2-cell embryos. FC denotes fold change; mKO denotes maternal knockout. **c** Overlap of candidate UHRF1-targeted TEs (upregulated in *Uhrf1*^*mat-KO*^ and downregulated in *Nlrp14*^*mat-KO*^ 2-cell embryos) and high-confident UHRF1 binding TEs in WT 2-cell embryos (identified in Supplementary Fig. S10b). **d** Line plots and heatmap showing the UHRF1 binding signals on four LTR subtypes in WT, *Nlrp14*^*mat-KO*^ and negative control (no injection) 2-cell embryos. The “*n*” number represents the total insertion of each LTR subtype in the genome. **e** Heatmap showing the paternal expression levels of four LTR subtypes in 2-cell embryos of WT and five mouse models generated in this study. **f** Box plots showing the paternal DNA methylation level (WCG methylation level) of four LTR subtypes in mouse sperm and 2-cell embryos from WT and five mouse models generated in this study. The diamond represents the mean WCG methylation level.

As UHRF1 was predominantly associated with TEs and primitive UBPs show strong enrichment at LTR elements, predominantly within the ERV1 and ERVK subfamilies (Figs. 2f, 5a), we subsequently focused on evaluating TE expression of paternal genome by integrating our multi-omics data. In *Uhrf1*^*mat-KO*^ 2-cell embryos, 109 subtypes of TEs were significantly upregulated, while in *Nlrp14*^*mat-KO*^ 2-cell embryos, 362 subtypes of TEs were significantly downregulated (Fig. 5b). Notably, 26 subtypes were shared between the two groups (Supplementary Fig. S10a), suggesting a potential association between these TEs and the turnover of UHRF1 localization. Peak-level annotation of primitive UBPs identified 11 high-confidence UHRF1-bound TE subtypes in WT embryos (Supplementary Fig. S10b). Intersecting these 11 TE subtypes with the 26 TE subtypes commonly dysregulated in both KO models yielded 4 overlapping TE subtypes: IAPLTR1a_Mm, RLTR27, IAPEz-int, and IAPEy-int (Fig. 5c), which therefore represent high-confidence candidates that are both functionally associated with UHRF1-dependent transcriptional regulation and directly bound by UHRF1. In contrast, there was no overlap among the TEs upregulated in *Nlrp14*^*mat-KO*^, downregulated in *Uhrf1*^*mat-KO*^, and bound by UHRF1 (Supplementary Fig. S10c), suggesting an indirect relationship with UHRF1. The robust detection of UHRF1 signals was observed in IAPLTR1a_Mm, RLTR27, IAPEz-int, and IAPEy-int in both WT and *Nlrp14*^*mat-KO*^ embryos (Fig. 5d; Supplementary Fig. S10d). These four LTR subtypes were inactive in the WT paternal genome but became activated in *Uhrf1*^*mat-KO*^ embryos and silenced in *Nlrp14*^mat-KO^ embryos (Fig. 5e). Notably, either the deletion of UHRF1 or inhibition of DNA methylation in *Nlrp14*^mat-KO^ embryos resulted in elevated activity of these LTRs (Fig. 5e). Furthermore, these four LTR subtypes evaded DNA demethylation in the paternal genome of WT embryos (Fig. 5f; Supplementary Table S8), in contrast to the global reduction of DNA methylation observed (Supplementary Fig. S10e and Table S8). Notably, in *Uhrf1*^*mat-KO*^ embryos, the aberrant activation of those TEs was accompanied by a slight increase in global chromatin accessibility in both parental genomes (Supplementary Fig. S10f and Table S8). Collectively, these findings suggest that the residual UHRF1 in the nuclei of WT embryos maintains specific LTR subtypes in an inactive state.

### Nuclear translocated UHRF1 silencing of LINE1 elements impedes ZGA

To further elucidate the relationship between aberrant nuclear localization of UHRF1 and impeded ZGA, we concentrated on TE subfamilies exhibiting enhanced UBP occupancy in *Nlrp14*^*mat-KO*^ embryos. In addition to retaining binding at a subset of LTR subfamilies that are already occupied in WT embryos, enhanced UBPs were significantly enriched at LINE1 subfamilies (Fig. 5a), particularly L1Md_A and L1Md_T (Supplementary Fig. S10b). Previous studies have demonstrated that the activation of LINE1, especially the evolutionarily young L1Md families, such as L1Md_A or L1Md_T, and the regulation of their RNA stability are causally associated with chromatin remodeling and ZGA^4,5,32^. Knockdown of LINE1 in mouse early embryos reduces global chromatin accessibility and impairs ZGA^4,5^. Despite initial documentation of the mechanism by which LINE1 regulates ZGA^5,33^, the regulatory mechanisms governing LINE1 itself in early embryos require further explorations.

**Fig. 6:**
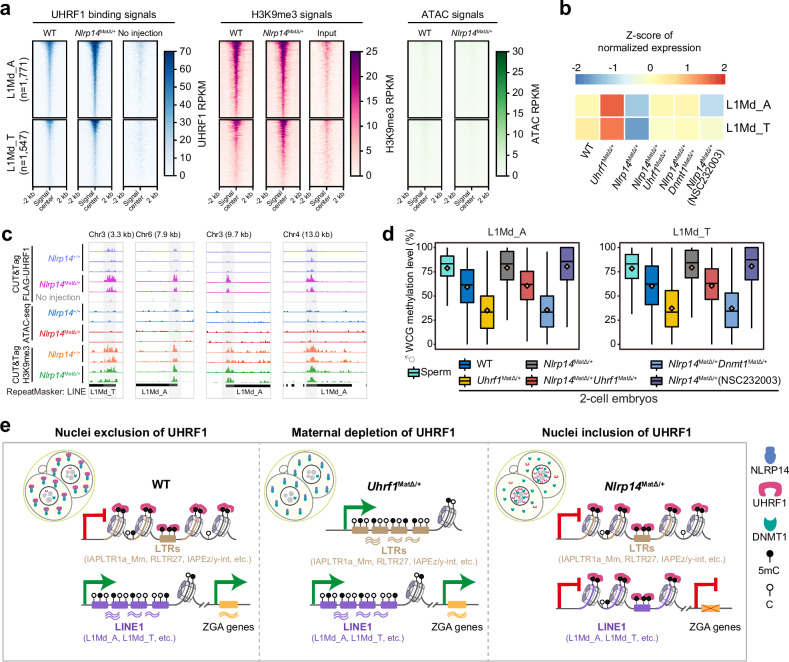
Nuclear translocated UHRF1 silences LINE1 elements in *Nlrp14*^*mat-KO*^ embryos. **a** Heatmaps showing the UHRF1 binding signals, H3K9me3 signals and ATAC-seq signals on a filtered set of LINE1 elements in WT and *Nlrp14*^*mat-KO*^ 2-cell embryos. The analysis includes 3318 LINE1 insertions that show detectable UHRF1 binding in *Nlrp14*^*mat-KO*^ 2-cell embryos. **b** Heatmap showing the expression levels of L1Md_A and L1Md_T elements in 2-cell embryos from WT and five mouse models generated in this study. **c** Track plot showing the representative UHRF1 targeted LINE1 loci. The signals of chromatin accessibility (ATAC) and H3K9me3 in these regions in WT and *Nlrp14*^*mat-KO*^ 2-cell embryos were also shown. The rectangular shadow box represents the UBP regions. Chromosomal coordinate information for the regions shown in this figure includes: Chr3: 149,746,589-149,749,864 (3.3 kb), Chr6: 107,992,172-108,000,060 (7.9 kb), Chr3: 145,525,150-145,534,864 (9.7 kb), Chr4: 25,651,204-25,664,409 (13.0 kb). **d** Box plots showing the paternal DNA methylation levels (WCG methylation levels) of L1Md_A and L1Md_T elements in mouse sperm and 2-cell embryos from WT and five mouse models generated in this study. The diamond represents the mean WCG methylation level. **e** A model illustrating the role of UHRF1 in early embryos. In WT embryos, DNMT1 and UHRF1 are predominantly localized in the cytoplasm, while residual UHRF1 in the nuclei maintains specific LTR subtypes in an inactive state in a DNA methylation-dependent manner. Depletion of UHRF1 (*Uhrf1*^*mat-KO*^) resulted in elevated activity of these LTRs. On the contrary, the nuclei inclusion of UHRF1 will silence these LTRs even more (*Nlrp14*^*mat-KO*^). Besides, aberrant nuclear localization leads to abnormal occupancy of UHRF1 and DNMT1 on LINE1 elements, prevents the demethylation process of LINE1 elements, and further silenced LINE1 then impedes ZGA.

Accordingly, we conducted a focused analysis of LINE1 elements exhibiting high-confidence UHRF1 binding and transcriptional inactivity in *Nlrp14*^*mat-KO*^ embryos (Fig. 6a–c). Strikingly, pronounced UHRF1 signals were detected within LINE1 elements in *Nlrp14*^*mat-KO*^ embryos; however, these bindings were markedly reduced or absent in WT embryos (Fig. 6a, c). Accordingly, these LINE1 elements, especially the mammalian-specific L1M families^34,35^, highlighted by the two longer L1Md_A and L1Md_T subtypes (Supplementary Fig. S10g), were activated in the WT but remained silenced in *Nlrp14*^*mat-KO*^ embryos (Fig. 6b). As expected, mat-KO of *Uhrf1* would not impede the activation of LINE1 in early embryos (Fig. 6b). Either deletion of UHRF1 through *Uhrf1/Nlrp14* DKO or reduction of DNA methylation via *Dnmt1/Nlrp14* DKO would reactivate LINE1 (Fig. 6b), suggesting that UHRF1 silences LINE1 through its association with DNA methylation. Moreover, the inhibition of UHRF1 binding through the application of NSC232003 in *Nlrp14*^*mat-KO*^ embryos resulted in the significant reactivation of L1Md_T (Fig. 6b). Why residual nuclear UHRF1 cannot bind to LINE1 as observed in LTR subtypes within WT embryos (Fig. 5)? To investigate this, we examined the DNA methylation dynamics in LINE1 elements in early embryos with varying genotypes or treatments (Fig. 6d; Supplementary Table S8). The examined LINE1 subtypes experienced significant DNA demethylation in both WT and *Uhrf1*^*mat-KO*^ embryos without exception (Fig. 6d). However, DNA demethylation was inhibited at these LINE1 elements in *Nlrp14*^*mat-KO*^ embryos (Fig. 6d). This inhibition was, however, rescued in *Dnmt1&Nlrp14*^*mat-DKO*^ embryos (Fig. 6d). Therefore, the nuclear exclusion of UHRF1 and DNMT1 ensures DNA demethylation in LINE1 elements, which in turn prevents residual nuclear UHRF1 in WT embryos from binding to LINE1. This mechanism leads to the activation of LINE1 and its subsequent role in ZGA and early embryonic development (Fig. 6e).

Finally, we conducted motif enrichment analysis to identify sequence features enriched within UHRF1-bound LTR subtypes (IAPLTR1a_Mm, RLTR27, IAPEz-int, and IAPEy-int) and LINE1 subtypes (L1Md_A and L1Md_T) in our dataset. Correspondingly, primitive UBPs identified in four LTR subtypes and enhanced UBPs identified in two LINE1 subtypes were selected for downstream analysis (Supplementary Fig. S10h). Notably, binding motifs for ZFP57, E2F4, and REST were significantly enriched in genomic regions associated with primitive UBPs in LTR subtypes and enhanced UBPs in LINE1 subtypes (Supplementary Fig. S10h). These factors are maternally expressed (Supplementary Fig. S10i) and previously characterized in the literature and functionally implicated in epigenetic silencing pathways^36–38^. Interestingly, primitive UBPs overlapping the four LTR subclasses showed specific enrichment for some factors such as OBOX2/5 and ZFP961 (Supplementary Fig. S10h, i), which were reported to recognize specific LTR subtypes or repress ERVs through recruitment of heterochromatin-inducing complexes^39,40^. In contrast, enhanced UBPs overlapping the two LINE1 subclasses also showed specific motif enrichment in several factors such as LEF1 and NR6A1 (Supplementary Fig. S10h, i). Whether these factors contribute to the differential recruitment of UHRF1 to LTR vs LINE1 subtypes remains further investigations. In summary, we have systematically revealed an important role of UHRF1 in early embryos and the biological significance of its nuclear exclusion in this study.

## Discussion

UHRF1 was originally identified as a novel methyl-CpG-binding protein (ICBP90) through EMSA followed by western blot analysis using cell extracts and fully methylated, rather than hemi-methylated DNA^41^. Later, UHRF1 (also known as NP95) was reported as a nuclear factor for DNMT1 that functions in the maintenance of DNA methylation in somatic and embryonic stem cells^28,42^. Conversely, UHRF1 was surprisingly abundantly detected in the cytoplasm but not in the nucleus in mature oocytes and preimplantation embryos, raising important questions about the regulatory mechanism and the biological importance of this unusual phenomenon^16,17^. The nuclear exclusion of UHRF1 in oocytes is achieved by active export through DPPA3 interaction and is needed for avoiding abnormal de novo DNA methylation^25^. However, DPPA3 localizes in the nucleus in early embryos that are spatially distinguished from UHRF1 (Supplementary Fig. S2)^25,43^, suggesting that a complementary mechanism substitutes DPPA3 function for direct UHRF1 localization. Instead, the NLRP14-controlled mechanism maintains UHRF1 in the cytoplasm of early embryos, and *Nlrp14* deficiency is accompanied by impeded ZGA and DNA demethylation. Interestingly, unlike DPPA3, the interaction between UHRF1 and NLRP14 is not conserved in somatic cells. These findings suggest that *Nlrp14*-deficient mice are valuable models for studying the biological importance of UHRF1 in the context of chromatin remodelling, epigenetic reprogramming and ZGA during OET.

ZGA in mice requires a series of corresponding TFs, which may serve as pioneer factors for chromatin remodelling^44^ or mediate the relocation of RNA polymerase II to chromatin^8^. Recent studies have unexpectedly revealed that TEs, particularly LINE1 and several LTR subtypes^5,45^, play a significant role in ZGA and developmental progression. However, while there is growing documentation regarding mammalian ZGA TFs and their direct associations with ZGA genes, the regulatory mechanisms governing ZGA TEs across the genome remain unclear due to their repetitive nature. Our findings demonstrated that the exclusion of UHRF1 from the nucleus prevents its excessive binding to TEs, especially LINE1 elements, after fertilization, thereby preserving LINE1 transcription to facilitate chromatin remodelling and subsequent activation of ZGA genes (Fig. 6e). Meanwhile, the residual UHRF1 that remains in a nuclear localization within zygotes binds to LTRs that evade DNA demethylation, thereby ensuring their inactivation. Therefore, this sophisticated mechanism serves as a “two-for-one solution” to regulate TEs activity across the entire genome at the onset of mammalian development. In contrast to ZGA TFs, which usually activate a limited subset of ZGA genes individually in mammalian early embryos, the nuclear inclusion of UHRF1 almost blocks the progression of the ZGA program (Fig. 6e). More importantly, UHRF1 is evolutionarily conserved in vertebrates and maternally inherited in both model organisms and humans^46,47^, suggesting the important role of regulating UHRF1 localization in early developmental processes.

How is UHRF1 differentially recruited to specific LTR vs LINE1 subtypes in WT and *Nlrp14* KO embryos? Our study focused on DNA methylation reprogramming during early embryonic development and further elucidated potential regulatory factors through motif enrichment analysis (Supplementary Fig. S10). Notably, motifs for ZFP57, E2F4, and REST were enriched in both LTR- and LINE1-associated UBP regions. These factors are previously characterized and closely linked to epigenetic silencing mechanisms. ZFP57 is a key regulator of genomic imprinting in mouse embryos and is required for maintaining DNA methylation at imprinting control regions^36^. E2F4 belongs to the repressive E2F family and forms transcriptional repressor complexes with pRB-related proteins and DRTF, recruiting histone deacetylases (HDACs) or SUV39H1 to establish repressive chromatin states^37^. REST has been reported to recruit CoREST and HDAC complexes to mediate chromatin compaction and gene silencing^38^. In contrast, primitive UBPs overlapping the four LTR subclasses showed specific enrichment for factors such as OBOX2/5 and ZFP961. OBOX2/5 are homeobox transcription factors, and members of this family have been reported to bind specific LTR subclasses^39^. ZFP961 belongs to the KRAB-ZFP family, a class of transcription factors well known for repressing ERVs through recruitment of heterochromatin-inducing complexes^40^. Enhanced UBPs overlapping the two LINE1 subclasses were specifically enriched for NR6A1 (GCNF), an essential developmental regulator that can physically recruit DNA methylation complexes and has been implicated in stable epigenetic silencing during differentiation^48^. Whether these factors contribute to the differential recruitment of UHRF1 to LTR versus LINE1 subtypes in early embryos, under physiological or pathological conditions, remains an open question warranting further investigation.

The question of whether DNA methylation reprogramming is causally linked to ZGA in mammals has long been debated, but the cytoplasmic localization of both UHRF1 and DNMT1 in early cleavage stage embryos has made it difficult to answer this question. The biological importance of DNA demethylation in early embryos cannot be elucidated through maternal KO of *Uhrf1* or *Dnmt1*, as these models demonstrate only the potential cytoplasmic function or maintenance of DNA methylation at imprints that are not essential for ZGA and preimplantation development^17,18^. The *Nlrp14*^*mat-KO*^ embryo serves as a gain-of-function model for exploring the causal relationship between DNA demethylation and ZGA by examining the roles of both UHRF1 and DNMT1 in the nucleus. Through the utilization of genetic DKO models, specifically *Uhrf1&Nlrp14*^*mat-DKO*^ and *Dnmt1&Nlrp14*^*mat-DKO*^ embryos, we discovered that excessive nuclear DNMT1 can maintain genome-wide DNA methylation independently of UHRF1 in zygotes and 2-cell embryos (Fig. 6e). Therefore, our findings refine the conventional belief that the limited nuclear UHRF1 only participates in maintaining DNA methylation in zygotes and 2-cell embryo stage as it does in somatic cells. Furthermore, epigenetic reprogramming was essential for facilitating LINE1 demethylation, thereby preventing the repression that could otherwise result from residual nuclear UHRF1 binding (Fig. 6e). Notably, paternal and maternal genomes undergo markedly distinct DNA methylation dynamics during mouse preimplantation development. Moreover, UHRF1 also plays a role in de novo DNA methylation during mouse oocyte growth^17^. In this study, we observed that DNA methylation at primitive and enhanced UBPs on the maternal allele increases from oocytes to 2-cell embryos in both WT and *Nlrp14*^*mat-KO*^ embryos. Interestingly, this increase is absent in *Uhrf1&Nlrp14*^*mat-DKO*^ embryos, suggesting that UHRF1 may contribute to de novo DNA methylation of the maternal genome during this transition. The asymmetric epigenetic reprogramming between parental genomes is intriguing and warrants more investigations. Overall, we present a distinctive model (Fig. 6e) elucidating the functional interplay and orchestration among DNA methylation reprogramming, retrotransposon activity regulation, and chromatin remodelling during mammalian ZGA and OET.

## Materials and methods

### Animal maintenance and KO strategies

WT C57BL/6 J mice were purchased from Vital River (China) and the Animal Center of the Institute of Zoology, Chinese Academy of Sciences. The PWK/PhJ strain and *Gdf9-iCre* transgenic mice were purchased from Jackson Laboratory (USA). The *Uhrf1*^*flox/flox*^ and *Dnmt1*^*flox/flox*^ mice were purchased from the Shanghai Model Organisms Center (China) and maintained on a C57BL/6J background. The *Dppa3* KO mice were purchased from GemPharmatech and maintained on a C57BL/6 J background. The *Nlrp14* KO mice were generated as described previously^22^. Male *Nlrp14*^*–/–*^ and female *Nlrp14*^*+/–*^ mice were mated to generate *Nlrp14*^*+/–*^ and *Nlrp14*^*–/–*^ offspring. Oocyte-specific deletion of UHRF1 or DNMT1 was achieved through the generation of [*Uhrf1*^*flox/flox*^, *Gdf9-iCre*] or [*Dnmt1*^*flox/flox*^, *Gdf9-iCre*] mice, respectively. For the *Nlrp14*^*–*/*–*^/*Uhrf1*^*–*/*–*^ double mutant oocytes, male [*Nlrp14*^*–/–*^, *Uhrf1*^*flox/flox*^, *Gdf9-iCre*] and female [*Nlrp14*^*+/–*^, *Uhrf1*^*flox/flox*^] mice were mated to generate female [*Nlrp14*^*–/–*^, *Uhrf1*^*flox/flox*^, *Gdf9-iCre*] mice. Similar breeding strategies were used to obtain mice with *Nlrp14*^*–*/*–*^/*Dnmt1*^*–*/*–*^ double mutant oocytes. For the *Nlrp14*^*V5-3×HA/+*^ knock-in mouse model, the V5-3×HA-linker element was inserted after the translation initiation site of the NLRP14-201 (ENSMUST00000084763.5) transcript by the CRISPR-Cas9 technique. The insertion element was ~0.1 kb in length and was expressed under the control of the endogenous NLRP14 gene. All animals were maintained and handled under the guidelines of the Institutional Animal Care and Use Committee of the Institute of Zoology of the Chinese Academy of Sciences.

### Cell culture and transfection

Human Embryonic Kidney 293 T (HEK293T) cells were cultured in Dulbecco’s modified Eagle’s medium (DMEM) supplemented with 10% FBS and 1% penicillin–streptomycin, and incubated at 37 °C in a humidified atmosphere containing 5% CO_2_. Transient overexpression of the exogenous gene in HEK293T cells was achieved by transfection with Lipofectamine-3000 reagent (Thermo Fisher Scientific). For the establishment of HEK293T cell lines stably expressing the FLAG-UHRF1-mCherry fusion protein, cells were transfected with the pBSR2-3×FLAG-UHRF1-mCherry plasmid using Lipofectamine-3000 reagent. Stable integrants were selected by culturing the cells in the presence of 10 μg/mL blasticidin-S-HCl (Thermo Fisher Scientific) for 7 days. Clonal cell lines were obtained by limiting dilution and validated by genotyping PCR, fluorescence microscopy, and western blotting. For leptomycin B treatment, 24 h after plasmid transfection, the cells were cultured in medium containing 20 nM leptomycin B for an additional 3 h before immunostaining.

### Plasmid construction

Coding sequences of *Nlrp14*, *Nlrp5*, *Uhrf1* and *Dppa3* were generated by PCR from cDNA of mouse MII oocytes. The full-length *Nlrp14* was cloned into the pcDNA3.1 expression vector with an N-terminal HA tag and a C-terminal FLAG tag. Truncated HA-tagged *Nlrp14* (aa 1–501) and FLAG-tagged *Nlrp14* (aa 493–933) plasmids were obtained by subcloning. *Nlrp5* and *Dppa3* were cloned into the pcDNA3.1 expression vector with either an N-terminal HA tag or an N-terminal V5 tag. The CDS of Enhanced Yellow Fluorescent Protein (EYFP) and *Uhrf1* were cloned into the pCMV expression vector with an N-terminal FLAG tag.

### Collection of mouse oocytes and early embryos

The growing oocytes were isolated from 15-day-old female mice. Germinal vesicle oocytes were isolated from 6- to 8-week-old female mice after 46 to 48 h of pregnant mare gonadotropin (PMSG) injection. Briefly, the ovaries were dissected and then digested in M2 medium containing 1 mg/mL collagenase IV for 20 min at 37 °C and 1000× rpm. Then, the ovarian tissues were transferred to 0.05% trypsin medium and incubated for another 20 minutes at 37 °C and 1000× rpm. The digested ovarian tissues were pipetted in M2 medium to dissociate the ovarian pieces into a cell suspension. After brief centrifugation to remove the supernatant, the cells were resuspended in M2 medium before manually picking oocytes under a stereomicroscope.

For the collection of mouse early embryos, 6- to 8-week-old female mice were superovulated and mated with 8- to 10-week-old PWK/PhJ or C57BL/6 J male mice. Zygotes were collected 20 h after hCG injection and cultured in mineral oil-covered drops of KSOM in a humidified atmosphere containing 5% CO_2_ and 90% N_2_. The PN3-5 zygotes, 2-cell embryos, and blastocysts were collected at 25 h, 48 h and 110 h post-hCG injection, respectively. For leptomycin B treatment, growing oocytes were isolated and cultured in KSOM containing 20 nM leptomycin B and 240 μM dibutyryl-cAMP for 3 h before immunostaining. For inhibitor-treatment experiments, WT and *Nlrp14*^*mat-KO*^ zygotes (24 h after hCG injection) were cultured in KSOM with the UHRF1 inhibitor NSC232002 (Selleck, Cat# E0351) at a final concentration of 500 μM. To evaluate the effect of UHRF1 inhibition in *Nlrp14*^*mat-KO*^ embryos on ZGA gene expression, following primers were used for RT-qPCR: *Nlrp14* (F-primer: GGAAACCGTGAGTGAGGACAA, R-primer: GGAGCCTCGGAAGTT-TGGATA); *Kdm5b* (F-primer: CTGGGAAGAGTTCGCGGAC, R-primer: CGCGGG-GTGAAATGAAGTTTAT); *Obox6* (F-primer: ATGCTTCAATACAATCAGAGCCC, R-primer: GGCAAATTCCTTGCAGGTTCTT); *Alppl2* (F-primer: GAGCGTCATCC-CAGTGGAG, R-primer: TAGCGGTTACTGTAGACACCC); *Zscan5b* (F-primer: ATGGGCAATACAGAAGATGGGC; R-primer: GGTCAAACCGGGACTTGTAAA).

### Immunofluorescence staining of cultured cells and mouse early embryos

For the HEK293T cells, cells were seeded on glass slides and harvested 24 h after the transfection of the exogenous gene plasmid. After washing with 1× DPBS, the cells were fixed with 4% PFA for 30 min at room temperature (RT) and then permeabilized with 0.2% Triton X-100 for 20 min before being blocked with 1% BSA for 30 min. After incubation with primary antibodies at 4 °C overnight, the cells were washed with 0.05% Tween-20 before being incubated with secondary antibodies for 1.5 h at RT. Finally, the cells were washed with 0.05% Tween-20 and mounted with Anti-Fade Mounting Reagent and DAPI (Thermo Fisher Scientific). Immunofluorescence detection of proteins in mouse zygotes and 2-cell embryos was performed following our previously published procedures^22^. For the blastocysts, the zona pellucida was first removed with Tyrode’s solution, and the embryos were subsequently fixed with 3.7% PFA for 1 h. The embryos were permeabilized with 0.2% Triton X-100 for 30 min and blocked in 3% BSA for 1 h at RT. Then, the embryos were incubated with primary antibodies at 4 °C overnight. After washing with 0.05% Tween-20, the embryos were incubated with secondary antibodies for 1.5 h at RT. Finally, the embryos were mounted and transferred to a confocal dish. All immunofluorescence images were captured using a Zeiss LSM-880 confocal microscope.

### Protein extraction and immunoprecipitation

For preparation of whole-cell lysates, transfected HEK293T cells were first washed with 1× DPBS and then incubated with lysis buffer (50 mM Tris-HCl, 150 mM NaCl, 1 mM EDTA, 1% NP40 and 5% glycerol) containing 1× Halt Protease Inhibitor Cocktail (Thermo Fisher Scientific) and 0.02 U/μL Cryonase Cold-Active Nuclease (TaKaRa) for 30 min on ice. After centrifugation at 13,000× *g* for 10 min at 4 °C, the supernatant was retained, and the protein concentration was determined using a BCA kit (Beyotime). The total cell lysate was then incubated with an antibody for 4 h at 4 °C, after which the mixture was added to prebalanced Protein A and Protein G agarose beads (Cytiva) and incubated overnight at 4 °C. The beads were then washed with lysis buffer before being boiled in 2× denaturing protein loading buffer (Solarbio) at 95 °C for 10 min. The supernatant was collected and used for Western blot analysis. For zygotes, the zona pellucida was first removed with Tyrode’s solution. A total of 500–1000 µL of protein was used for co-IP as described above. The co-IP products were subjected to mass spectrometry or western blotting to identify proteins associated with the selected target proteins.

### Immunoblotting assay

Immunoblotting assays of extracted protein samples and IP products were performed using traditional Western blot methods and capillary immunoassays. For the capillary immunoassay, samples were processed and analysed according to the instructions. For traditional Western blotting, the samples were denatured at 95 °C for 10 min in protein loading buffer (Solarbio, Cat# P1040), loaded on 8%, 10%, and 15% SDS–PAGE gels, and semi-dry electroblotted onto PVDF membranes. Finally, the membrane was probed with primary antibodies (listed below) at 4 °C overnight and incubated with secondary antibodies against rabbit IgG (horseradish peroxidase-conjugated; Proteintech, Cat# SA00001-2) or mouse IgG (horseradish peroxidase-conjugated; Proteintech, Cat# SA00001-1) at RT for 1.5 h. The chemiluminescence signal was detected using Western ECL peroxide buffer (Tanon, Cat# 180-501 W) and luminol/enhancer solution (Tanon, Cat# 180-501B).

### Antibody information

The primary antibodies used in this study were as follows: anti-NLRP14 rabbit polyclonal antibody (homemade); anti-DNMT1 (Cell Signaling, Cat# 5032S); anti-UHRF1 (Santa Cruz, Cat# sc-373750); anti-UHRF1 (ABclonal, Cat# A2343); anti-DPPA3 (Abclonal, Cat# A20176); anti-HA (ABclonal, Cat# AE008); anti-V5 (ABclonal, Cat# AE017); anti-IgG (Abclonal, Cat# AC011); anti-FLAG (Sigma, Cat# F1804); anti-NLRP5 (Abmart, Cat# M034859); anti-H3K9me3 (Active Motif, Cat# 39161); anti-OCT4 (Santa Cruz, Cat# sc5279); anti-GATA6 (R&D Systems, Cat# AF1700); anti-CDX2 (Cell Signaling, Cat# 12306S); and anti-GAPDH (Cell Signaling, Cat# 2118); anti-β-actin (Proteintech, Cat# HRP-60008). The secondary antibodies used in this study were as follows: goat anti-mouse Alexa Fluor 488 (Invitrogen, Cat# A11029); goat anti-rabbit Alexa Fluor 555 (Invitrogen, Cat# A21429); donkey anti-mouse Alexa Fluor 488 (Invitrogen, Cat# A21202); donkey anti-goat Alexa Fluor 568 (Invitrogen, Cat# A11057); and donkey anti-rabbit Alexa Fluor 647 (Invitrogen, Cat# A31573).

### Haematoxylin-eosin staining

Testes or ovaries were dissected and then fixed in 4% PFA for 24 h. The fixed testes or ovaries were stored in 70% ethanol (EtOH) before being embedded in paraffin. The embedded testes or ovaries were cut into 5-μm thick sections and attached to pretreated microscope glass slides with enhanced tissue adhesion. After consecutive treatment with xylene, 100% EtOH, 95% EtOH, 70% EtOH, and distilled H_2_O, the deparaffinized sections were processed for haematoxylin-eosin staining. Briefly, the sections were stained with haematoxylin solution for 1 min, rinsed in running tap water for 15 min, and then placed in distilled H_2_O for 30 s and 95% EtOH for another 30 s. The tissue sections were further counterstained with eosin Y solution for 1 min, dehydrated, and cleared by consecutive treatment with 95% EtOH, 100% EtOH, and xylene. Finally, the tissue sections were dried at 37 °C for 2 days before imaging.

### RNA-seq of mouse zygotes and 2-cell embryos

The zona pellucida of the mouse zygote or 2-cell embryo was first removed using Tyrode’s solution (Sigma), followed by washing three times in DPBS supplemented with 0.04% BSA. Next, a single embryo was picked and transferred to the lysis buffer for RNA-seq library construction using the Smart-seq2 method. In brief, the cell membrane was permeabilized with Triton X-100 (Sigma) in the lysis buffer, and poly(A) + RNA was captured using oligo(dT) primers. First-strand cDNA was synthesized by SuperScript II reverse transcriptase (Thermo Fisher Scientific) during the reverse transcription process. The double-strand cDNA was then amplified, purified and fragmented to construct the RNA-seq library by using the NEBNext UltraII DNA Library Prep Kit (New England Biolabs, Cat# E7645L) according to the manufacturer’s protocol. After quality control using the Fragment Analyzer, the final libraries were sequenced on an Illumina NovaSeq 6000 sequencer with a 150 bp paired-end sequencing strategy.

### scChaRM-seq of mouse 2-cell embryos

First, the zona pellucida-removed single mouse 2-cell embryos were picked into a 250-µL PCR tube containing lysis buffer. In vitro GpC methylation was then performed to capture the chromatin accessibility information. Next, the cell and nuclear membranes were fully lysed, and mRNA was extracted using magnetic streptavidin beads (Thermo Fisher Scientific), following our previously reported RNA-seq library construction procedure^29^. The corresponding genomic DNA was bisulfite converted and subjected to library construction according to the tailing- and ligation-free method for single cells (TAILS) protocol^30^. The DNA and RNA libraries were sequenced on a NovaSeq 6000 sequencer with a 150-bp paired-end sequencing strategy.

### Construction of the WGBS libraries

Mouse 2-cell embryos (10 embryos pooled as one biological replicate) were subjected to bisulfite conversion and purification using the EZ-96 DNA Methylation-Direct MagPrep kit (Zymo Research, Cat# D5045), following the manufacturer’s instructions. Whole-Genome Bisulfite Sequencing (WGBS) libraries were then constructed using the TAILS method. Briefly, the first round of random priming was performed with P5-N6-oligo1 (5′-CTACACGACGCTCTTCC-GATCTN_6_-3′) in the presence of the Klenow exo(-) fragment (Qiagen, Cat# P7010-HC-L). Exo-SAP IT Express (Applied Biosystems, Cat# 75001) was used to remove the remaining oligos and dNTPs, and dC tailing was carried out using the TdT enzyme (Thermo Fisher, Cat# EP0162). A second round of priming was then performed using P7-G6-oligo2 (5′-AGACGTGTGCTCTTCCGATCTG_6_HN-3′) in the presence of the Klenow exo(-) fragment. After one round of purification with AMPure XP beads, libraries were constructed via PCR amplification. The libraries were then purified, and the size distribution was assessed on the fragment analyzer. Finally, the WGBS libraries were sequenced on a NovaSeq 6000 sequencer with a 150-bp paired-end sequencing strategy.

### ATAC-seq of HEK293T cells and mouse 2-cell embryos

HEK293T cells or mouse 2-cell embryos were directly transferred into 6 μL of lysis buffer containing 10 mM Tris-HCl, 10 mM NaCl, 3 mM MgCl_2_ and 0.5% NP-40. The mixture was pipetted to ensure thorough mixing and incubate on ice for 10 min. Next, 4 μL of 5× TTBL, 1 μL of homemade Tn5 and 9 μL of nuclease-free water were added to the permeabilized sample without removing the lysis buffer. The tagmentation reaction was mixed and incubated at 37 °C for 30 min. After the reaction, DNA was solubilized by adding 10% SDS and incubating at 55 °C for 10 min. The tagmented gDNA fragments were then purified and amplified in 1× Fidelity buffer, supplemented with 0.3 mM dNTPs, 1 U of KAPA HiFi DNA polymerase and 400 nM of forward and reverse index primers. AMPure XP beads were used for size selection of the final libraries. Finally, the size distribution of the ATAC library was assessed using a Fragment analyzer, and sequencing was performed on a NovaSeq 6000 sequencer with a 150-bp paired-end sequencing strategy.

### In vitro transcription and microinjection

First, the in vitro transcription template was amplified from the FLAG-UHRF1-HA plasmid using PrimeSTAR Max DNA Polymerase (Takara). mRNA was then synthesized using the mMESSAGE mMACHINE T7 Ultra Kit (Thermo Fisher Scientific, Cat# AM1345) according to the manufacturer’s instructions. Next, mRNA was purified using the MEGAclear Kit (Thermo Fisher Scientific, Cat# AM1908) and eluted with nuclease-free water. For mRNA microinjection, mouse MII oocytes were injected with approximately 10 pL of mouse FLAG-*Uhrf1*-HA mRNA using a Femtojet microinjector (Eppendorf) set to a constant flow setting. The embryos were then obtained through intracytoplasmic sperm injection (ICSI) using fresh sperm from PWK/PhJ male mice. The control and injected embryos were cultured until day 2 for CUT&Tag of FLAG-UHRF1.

### CUT&Tag of HEK293T cells and mouse 2-cell embryos

CUT&Tag libraries were constructed according to the manufacturer’s protocol for the Hyperactive Universal CUT&Tag Assay Kit for Illumina Pro (Vazyme, Cat# TD904). For HEK293T cell samples initiated with 100 k cells, the cell suspension was centrifuged at room temperature for 5 min at 600× *g*, followed by washing the cell pellet in 500 μL of wash buffer. The pellet was then centrifuged again and resuspended in 100 μL of wash buffer. For HEK293T cells samples with 500 cells and mouse 2-cell embryos, the samples were directly transferred into 100 μL of wash buffer. Activated Concanavalin A-coated magnetic beads were added to each sample, followed by a 10-min incubation at room temperature. After this, place the sample tube on a magnetic rack, discarded the supernatant once the solution cleared. Immediately afterward, the samples were resuspended in 50 μL of cooled antibody buffer. Next, primary antibody was added, the tubes were inverted to mix, and the samples were incubated on a rotating platform for 12 h at 4 °C. Then, discarded the supernatant and add the second antibody in Dig-wash buffer to each tube, incubate on a rotating platform for 1 h at RT. After antibody incubation, embryos were washed three times with Dig-Wash buffer. pA/G-Tnp incubation followed, taking place on a rotating platform for 1 h at room temperature. To remove unbound pA/G-Tnp protein, the embryos were washed three times with Dig-300 buffer and resuspended in tagmentation buffer. The samples were incubated at 37 °C for 1 h. Following tagmentation, 10% SDS was added to halt the reaction, and the samples were incubated at 55 °C for 10 min. Soon afterwards, the samples were plated on a magnetic rack, and the supernatant was carefully transferred to a new PCR tube. DNA was extracted using DNA Extraction Beads Pro. Finally, libraries were constructed using the TruePrep Index Kit V2 for Illumina (Vazyme, Cat# TD202). All CUT&Tag libraries were sequenced on the Illumina NovaSeq 6000 platform with a PE150 sequencing strategy. The primary and secondary antibodies used for CnT were as follows: anti-FLAG (Sigma, Cat# F1804); anti-H3K9me3 (Active Motif, Cat# 39161); anti-rabbit IgG (Abcam, Cat# ab171870); donkey anti-mouse (CST, Cat# 52885), and goat anti-rabbit (CST, Cat# 35401).

### Data processing

#### Quantifying the expression in RNA-seq data

The raw sequencing reads generated from scChaRM-seq and Smart-seq2 were trimmed with Trim Galore (v0.6.6). Alignment was performed using STAR (v2.7.10b), with the UCSC version mm9 genome sequence used as the reference. The raw gene expression data were quantified using featureCounts (v2.0.4). To annotate reads to TEs, mapping was done by STAR (v2.7.10b) with two parameters: *--outFilterMultimapNmax 100 and --outAnchorMultimapNmax 100*. Then TEs were counted using TEcount (v2.2.3). The GTF file for gene and TE annotation was retrieved from the UCSC Genome Browser.

### Processing the WGBS data

The raw reads were removed 9 bases from the 5′ end, and the low-quality and adapter-contaminated reads were discarded using Trim Galore (v0.6.6). The cleaned reads were first aligned to the in silico bisulfite-converted mouse genome reference (mm9) using Bismark (v0.23.1) in paired-end mode. Then, the unmapped reads from the first mapping were then re-aligned to the same reference in single-end mode. The bam files were merged, and duplicated reads were removed using SAMtools (v1.21). Finally, per-base methylation metrics were extracted from the cleaned bam file using MethylDackel (v0.6.1).

### Processing the scChaRM-seq data

The processing of the scChaRM-seq data was performed as previously described^29,30^. To remove sequencing adapters and low-quality reads, raw 150-bp paired-end reads were processed using TrimGalore (v0.6.6) with the following parameters: *--quality 20 --stringency 3 --length 50 --clip_R1 9 --clip_R2 9 --paired --trim1 --phred33*. The clean reads were then aligned to the reference genome (UCSC version mm9) using Bismark (v0.23.1). The alignment process was initially carried out in paired-end mode, followed by two independent rounds of alignment in single-end mode for unmapped Read#1 and Read#2. PCR duplicates were removed using SAMtools (v1.21). The methylation levels of the WCG (W = A or T) and GCH (H = A, T or C) sites were determined by MethylDackel (v0.6.1).

### Processing the ATAC-seq data

For ATAC-seq data, adapter sequences and low-quality reads were removed from the raw 150-bp paired-end reads using TrimGalore (v0.6.6). The clean reads were aligned to the reference genome (mm9 or hg19) using Bowtie2 (v2.4.5) with the following parameters: *--local --very sensitive --no-mixed --no-discordant -X 2000*. PCR duplicates were removed using sambamba (v0.8.2), and unmapped reads were removed using SAMtools (v1.21). Only uniquely mapped reads were used for further analysis. The reads were further shifted to + 4 bp and –5 bp for the positive and negative strands, respectively. The TSS enrichment score was calculated using the R package ChrAccR (v0.9.21).

### Processing of CUT&Tag and ChIP-seq data

The raw sequencing reads were trimmed to remove adapters using Trim Galore (v0.6.6). Clean reads were further aligned to the reference genome (UCSC version mm9) using Bowtie2 (v2.4.5) with the following parameters: *--local --very sensitive --no-mixed --no-discordant --phred33 -I 10 -X 700*. PCR duplicates were identified and removed using *sambamba* (v0.8.2). Unmapped reads were excluded using SAMtools (v1.21). Only uniquely mapped reads were used for further analysis. Read pairs that were on the same chromosome and had fragment lengths less than 1000 bp were used for downstream analysis.

### Peak calling

For ATAC-seq, peaks were first identified using MACS2 (v2.2.6) with a *P*-value cutoff of 0.01. Then, peaks identified in both replicates and greater than 100 bp in length were retained. Additionally, only peaks with an RPKM greater than 50 and RPKM difference of less than 2-fold between replicates were kept. For H3K9me3, peak calling was performed using SEACR (v1.3) with the following parameters: *0.01 non stringent*, and peaks identified in all replicates were retained. For UHRF1, peaks were first identified using SEACR (v1.3) by selecting the top 1% of regions based on the area under the curve (AUC). Peaks called in all replicates and with an RPKM greater than that of the Input group were used for subsequent analysis. Generally, any peaks that overlapped with the ENCODE mm9 blacklist were excluded.

### Permutation-based enrichment analysis of UBPs at TEs

Permutation tests were performed to assess the enrichment of UBPs at TE families, including SINE, LINE, and LTR. TE annotations were obtained from the mm9 genome and grouped by TE subclass. All annotated TE insertions belonging to the same class were merged to generate non-overlapping genomic feature intervals. For each TE class, the observed overlap was calculated as the total base-pair length shared between UBP regions and the corresponding TE intervals using bedtools intersect (v2.30.0). To establish a null distribution, randomized genomic regions were generated using bedtools shuffle (v2.30.0), while preserving both the number and length of the original UBP regions and excluding blacklisted genomic regions. A total of 10,000 permutations were performed for each analysis. For each permutation, the total overlap length between the shuffled regions and the corresponding TE intervals was calculated. Enrichment was quantified as an odds ratio, defined as the observed overlap divided by the mean overlap obtained from the randomized permutations. Empirical *P* values were calculated as (k + 1)/(N + 1), where k represents the number of permutations with overlap greater than or equal to the observed value and N is the total number of permutations (10,000).

### Motif enrichment analysis at UHRF1-bound TEs

Primitive UBPs (pUBPs) and enhanced UBPs (eUBPs) were intersected with RepeatMasker annotations of specific TE subfamilies. For LTR analyses, UBPs overlapping IAPLTR1a_Mm, RLTR27, IAPEz-int, or IAPEy-int were identified. For each overlap, the region with the highest signal intensity on the peak was selected, and a 200-bp window centered on the region was extracted for motif analysis. This generated pUBP w/ LTR sequences. For LINE1 analyses, enhanced UBPs overlapping L1Md_A or L1Md_T were identified and regions were extracted using the same criteria, yielding the eUBP w/ LINE1 sequences. All downstream motif analyses were performed on these 200-bp sequences. To control for TE class and sequence composition bias, background regions were generated separately for each target dataset. For pUBP w/ LTR sequences, background regions were sampled from ERV1 and ERVK LTR subfamilies excluding IAPLTR1a_Mm, RLTR27, IAPEz-int and IAPEy-int. For eUBP w/ LINE1, background regions were sampled from LINE1 subfamilies excluding L1Md_A and L1Md_T. Background regions were matched to target regions in number, length, and GC content distribution to minimize confounding due to sequence composition. Motif enrichment was assessed using Simple Enrichment Analysis (SEA) from the MEME Suite (v5.5.9). Enrichment statistics were calculated using default SEA parameters. Enriched motifs were filtered based on mouse zygote RNA-seq expression data. Only genes with expression levels within the top 50%, enrichment score > 2 and E-value < 1 × 10^–^¹⁰ were retained.

### Calculation of the Pearson correlation coefficient

To assess the correlation between different replicates in ATAC-seq, CUT&Tag and ChIP-seq data, the Pearson correlation coefficient was calculated. Briefly, the genome was first divided into 5-kb bins, and the peak positions were used for H3K9me3 CUT&Tag. Read coverage was obtained using *deeptools multiBamSummary*. The raw counts were normalized to RPKM. Pearson’s correlation was then computed using R (v4.1.2), and the results were visualized using either pheatmap (1.0.12) or ggplot2 (v3.3.5).

### Differential UHRF1 binding peaks between WT and *Nlrp14*^*mat-KO*^ 2-cell embryos

Clean peaks from WT and *Nlrp14*^*mat-K****O***^ 2-cell embryos were merged into a new set using the reduce function in the GenomicRanges package. For each genotype, the average RPKM of these peaks was calculated from the corresponding BAM files. Peaks were then classified based on the *Nlrp14*^*mat-KO*^-to-WT average RPKM ratio: primitive UBP (0.8 ≤ ratio < 1.2) and enhanced UBP (ratio ≥ 1.2). For peaks annotation, TSS and transcription end site (TES) information was obtained from the UCSC Genome Browser (mm9), and all repetitive elements, including their subfamilies, were annotated from RepeatMasker. Promoters were defined as regions from –3 kb to +3 kb relative to the TSS, while genebodies were defined as regions from the TSS to the TES. The complementary regions of genebodies in the genome were defined as intergenic regions.

### Identification of allele-specific alignments from sequencing data

SNPsplit (v0.5.0) was used to identify allele-specific alignments for the NGS data as previously described. Briefly, the clean reads were aligned to the N-masked mm9 mouse genome, which was generated based on all known PWK-PhJ SNP information (Mouse Genomes Project). The aligned reads were further grouped into maternal (C57BL/6J) or paternal (PWK-PhJ) reads for further analysis. A customized in-house algorithm derived from SNPsplit was used to discern parental-specific reads within the long-read dataset. Briefly, clean reads were aligned to the N-masked reference genome, and for each mapped read, the algorithm quantified the number of SNP sites and SNP sites featuring deletions (N-deletions at known SNP sites). Reads with more than 2 N-deletions at known SNP sites were excluded from further analysis. The remaining reads underwent additional refinement. Each N within the reads was examined and categorized into maternal or paternal known SNP sites and unknown sites. The majority rule was applied to identify allele-specific reads utilizing the following indicator formula: (# maternal SNP sites - # paternal SNP sites - # N-deletion SNP sites)/# SNP sites. A read was considered maternal specific if the indicator exceeded 0.5. Conversely, if the indicator fell below –0.5, the read was classified as paternal specific.

### ZGA genes

ZGA genes were defined and identified based on a published dataset and specific expression criteria as previously described^8^. ZGA genes were characterized by their expression patterns: they were either unexpressed or minimally expressed in full-growth and MII oocytes (with an FPKM < 5) but showed a significant increase in expression (FPKM > 5 with a minimum threefold increase) in both 1-cell and early 2-cell embryos, defining them as minor ZGA genes. Alternatively, if the genes were expressed in oocytes (FPKM > 5) and demonstrated a substantial increase in expression at the late 2-cell stage (more than fivefold), they were classified as major ZGA genes.

### Statistical analyses

Statistical analyses were performed in R (v4.1.2). Differences between the two groups were assessed using *t*-tests to evaluate whether their means differed significantly. Effect sizes were quantified using Cohen’s d, calculated with the effsize package (v0.8.1). For comparing the primitive UBPs to the enhanced UBPs, statistical significance was evaluated using Fisher’s exact test as implemented in the R function *fisher.test*.

## Supplementary information

Supplementary Figs. S1-S10

Supplementary Table S1

Supplementary Table S2

Supplementary Table S3

Supplementary Table S4

Supplementary Table S5

Supplementary Table S6

Supplementary Table S7

Supplementary Table S8

## Data Availability

Raw and processed data generated in this study was deposited in the GSA (Genome Sequence Archive) under the accession number CRA014086 in the National Genomics Data Center (NGDC, https://bigd.big.ac.cn/). The following datasets included in this study are available via the NCBI GEO database: Smart-seq2 and scCOOL-seq data of WT and *Nlrp14*^*mat-KO*^ 2-cell embryos (GSE186357); scRNA-seq data of mouse early embryos (GSE45719); scCOOL-seq data of mouse sperm and oocytes (GSE78140); ATAC-seq data of mouse 2-cell embryos (GSE196520 and GSE66390); ULI-NChIP-seq data of H3K9me3 in mouse 2-cell embryos (GSE97778).
