# Supplementary material for: Selective cellular localization of UHRF1 safeguards mammalian zygotic genome activation and early embryonic development
Source: Cell Discov. 2026 May 26;12:38. doi: 10.1038/s41421-026-00896-3 (PMC13212597; doi:10.1038/s41421-026-00896-3)
Supplement: Supplementary file 1 — Supplementary Figs. S1-S10 [file 41421_2026_896_MOESM1_ESM.pdf]

Supplementary Information

Selective cellular localization of UHRF1 safeguards mammalian zygotic genome activation and early embryonic development

Rui Yan, Xin Cheng, Xin Long, Yating Zhu, Qiancheng Zhang, Fengyuan Sun, Fan Zhang, Mengyue Wang, Ruifeng Zhang, Tianzi Guo, Xinling Hou, Dongmei Ji, Yunxia Cao, Fei Gao, Dan Liang, Fan Guo

The file includes Supplementary Figs. S1-S10.

Other Supplementary Information for this manuscript includes Supplementary Tables S1-S8.

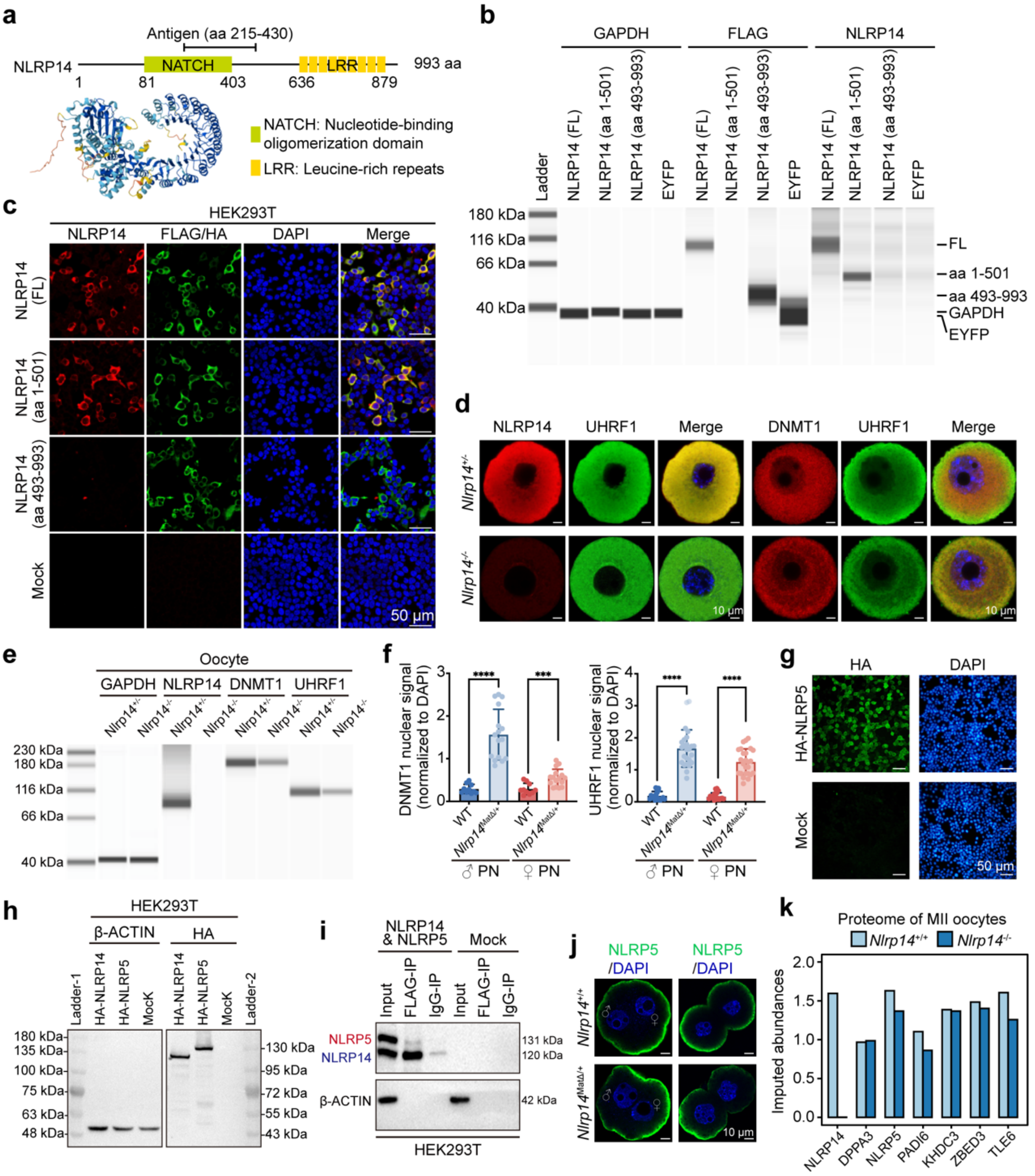

Supplementary Fig. S1 | Homemade antibody validation and relationships of NLRP14 with SCMC members. a, Protein structure and domains of NLRP14 predicted by AlphaFold-2. Location of the antigen region (aa 215–430) is indicated. **b-c,** Evaluation of the NLRP14 antibody through ectopic expression of full-length or truncated HA tagged NLRP14 (aa 1-501) and FLAG tagged NLRP14 (aa 493-933) protein in HEK293T cells. Antibody detection was performed by WES (**b**) and immunofluorescence (**c**) assays. **d,** Immunofluorescence staining of NLRP14, UHRF1 and DNMT1 in WT and *Nlrp14* KO GV oocytes. For NLRP14 and UHRF1 co-staining: WT, n=8; *Nlrp14*$^{-/-}$, n=8. For DNMT1 and UHRF1 co-staining: WT, n=8;

Nlrp14$^{-/-}$, n=8. **e,** WES was used to determine the protein expression levels of NLRP14, UHRF1 and DNMT1 in WT and *Nlrp14* GV KO oocytes. GAPDH was used as the internal control. **f,** Quantification of DNMT1 and UHRF1 signal intensity in male and female pronuclei of WT and *Nlrp14*$^{mat\text{-}KO}$ embryos, normalized to DAPI. Statistical significance was assessed using a two-tailed Student's *t*-test. For DNMT1: WT, n = 10; *Nlrp14*$^{mat\text{-}KO}$, n = 16. For UHRF1: WT, n = 18; *Nlrp14*$^{mat\text{-}KO}$, n = 25. **g,** Immunofluorescence staining of HA in HA-NLRP5 overexpressed HEK293T cells. **h,** Western blotting confirmed the overexpression of HA-NLRP14 or HA-NLRP5 in HEK293T cells via the use of an HA antibody. β-ACTIN was used as the internal control. **i,** Western blot analysis of HA-NLRP5 and HA-NLRP14 in HEK293T cells following FLAG co-IP. FLAG was tagged on the C-terminus of NLRP14. **j,** Immunofluorescence staining of NLRP5 in WT and *Nlrp14*$^{mat\text{-}KO}$ zygotes (WT, n = 3; KO, n = 4) and 2-cell (WT, n = 3; KO, n = 3) embryos. **k,** Bar plot showing the imputed abundances of NLRP14, DPPA3, NLRP5, PADI6, KHDC3, ZBED3 and TLE6 in WT and *Nlrp14* KO MII oocytes.

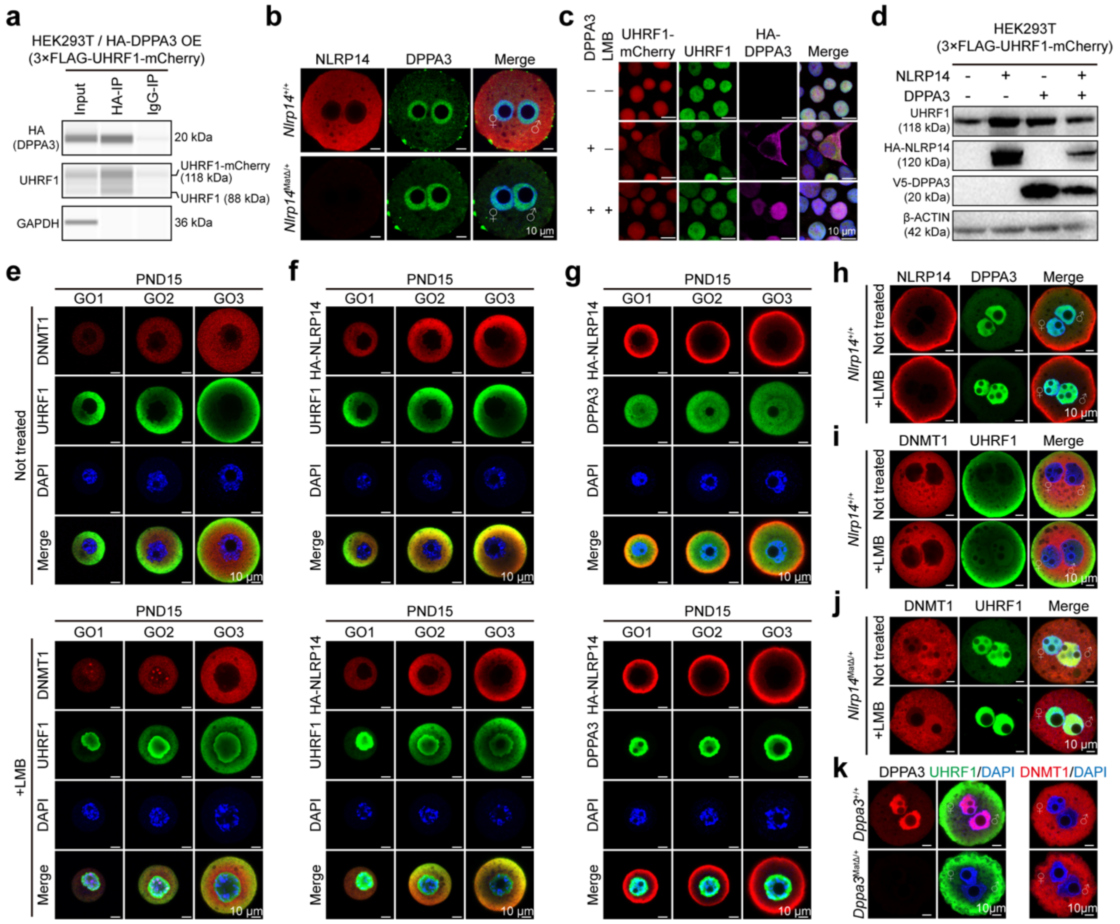

Supplementary Fig. S2 | Subcellular localization of NLRP14, DPPA3, UHRF1 and DNMT1 in mouse growing oocytes and zygotes. a, Co-immunoprecipitation (co-IP) was used to detect the interaction between UHRF1 and DPPA3 with an HA antibody after transient overexpression of HA-DPPA3 in 3×FLAG-UHRF1-mCherry HEK293T cells (with stable overexpression of 3×FLAG-UHRF1-mCherry). The interaction between DPPA3 and UHRF1 was detected using the WES. **b,** Immunofluorescence staining of NLRP14 and DPPA3 in WT (n = 4) and *Nlrp14*$^{mat\text{-}KO}$ (n = 3) zygotes. **c,** Immunofluorescence staining of UHRF1 and HA-DPPA3 in WT and DPPA3 OE HEK293T cells, with or without LMB treatment. **d,** Western blotting for UHRF1, NLRP14, and DPPA3 in 3×FLAG-UHRF1-mCherry HEK293T cells ectopically expressing NLRP14 or DPPA3. **e-g,** Immunofluorescence staining for the subcellular localization of DNMT1 and UHRF1 (**e**), NLRP14 and UHRF1 (**f**), NLRP14 and DPPA3 (**g**) in growing oocytes (GO) with or without LMB treatment. For DNMT1 and UHRF1 co-staining: GO1-GO3 oocytes were analyzed separately

(Not treated, n = 5 per stage; with LMB, n = 5 per stage). For NLRP14 and UHRF1 co-staining: GO1-GO3 oocytes were analyzed separately (Not treated, n = 3 per stage; with LMB, n = 4 per stage). For NLRP14 and DPPA3 co-staining: GO1-GO3 oocytes were analyzed separately (Not treated, n = 10 per stage; with LMB, n = 10 per stage). PND, Postnatal Day. **h-j,** Immunofluorescence staining for examining the subcellular localization of NLRP14 and DPPA3 (**h**), DNMT1 and UHRF1 (**i**) in WT zygotes treated with or without LMB, and DNMT1 and UHRF1 in *Nlrp14*$^{mat\text{-}KO}$ zygotes treated with or without LMB (**j**). For NLRP14 and DPPA3 co-staining: Not treated, n = 10; with LMB, n = 8. For DNMT1 and UHRF1 co-staining in WT zygotes: Not treated, n = 6; with LMB, n = 6. For DNMT1 and UHRF1 co-staining in *Nlrp14*$^{mat\text{-}KO}$ zygotes: Not treated, n = 6; with LMB, n = 5. **k,** Immunofluorescence staining of DPPA3, UHRF1 and DNMT1 in WT and *Dppa3*$^{mat\text{-}KO}$ zygotes. For UHRF1 and DPPA3 co-staining: WT, n = 4; KO, n = 5. For DNMT1 staining: WT, n = 5; KO n = 5.

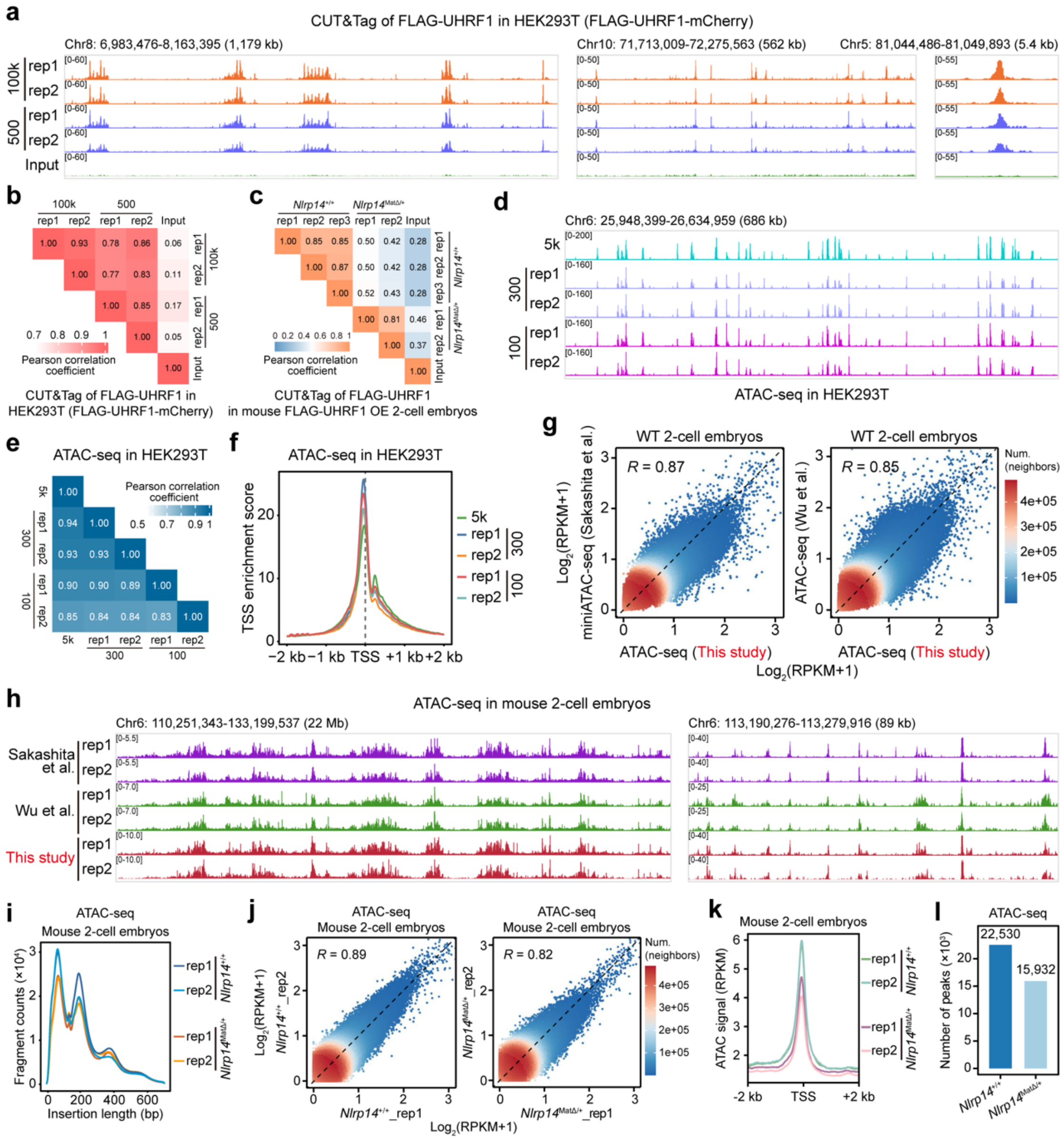

Supplementary Fig. S3 | Quality control of FLAG-UHRF1 CUT&Tag and ATAC-seq data in HEK293T cells and mouse 2-cell embryos. a, Track plot showing the FLAG-UHRF1 binding signals in FLAG-UHRF1-mCherry knock-in HEK293T cells, and CnT of FLAG-UHRF1 in WT HEK293T cells was the negative control. **b,** Heatmap showing Pearson correlation coefficients of UHRF1 signal intensity across UBPs in FLAG-UHRF1-mCherry stably overexpressing HEK293T cells prepared from different input cell numbers. WT HEK293T cells were used as input control. **c,** Heatmap showing Pearson correlation coefficients of UHRF1 signal intensity across UBPs in FLAG-UHRF1 overexpressing WT and *Nlrp14*$^{mat\text{-}KO}$ 2-cell embryos. Non injection 2-cell embryos were used as input control. **d,** Track plot

showing chromatin accessibility in HEK293T cells with different cell amounts. **e,** Heatmap showing the Pearson correlation coefficient of chromatin accessibility in down-sampled HEK293T cells. The genome was divided into 5-kb tiles, and the ATAC signal was shown in log_2(RPKM+1) values. **f,** TSS enrichment score indicating chromatin accessibility in HEK293T cells. **g,** Scatter plots showing the consistency (Pearson correlation values, R) of ATAC-seq data generated in this study and published data (Sakashita A et al., Nature Genetics, 2023; Wu et al., Nature, 2016) in mouse WT 2-cell embryos. Enrichment per 5-kb bin was shown in log_2(RPKM+1) values. **h,** Track plot showing chromatin accessibility in WT 2-cell embryos. Published WT 2-cell ATAC-seq data were used to evaluate data consistency. **i,** Line plot showing the length distributions of fragments captured by ATAC-seq in WT and *Nlrp14*$^{mat\text{-}KO}$ 2-cell embryos. **j,** Scatter plots showing the reproducibility (Pearson correlation values, R) of ATAC-seq data between two biological replicates. Enrichment per 5-kb bin was shown in log_2(RPKM+1) values. **k,** Chromatin accessibility of the TSS (from 2-kb upstream to 2-kb downstream) in WT and *Nlrp14*$^{mat\text{-}KO}$ 2-cell embryos. **l,** The number of ATAC-seq peaks in WT and *Nlrp14*$^{mat\text{-}KO}$ 2-cell embryos.

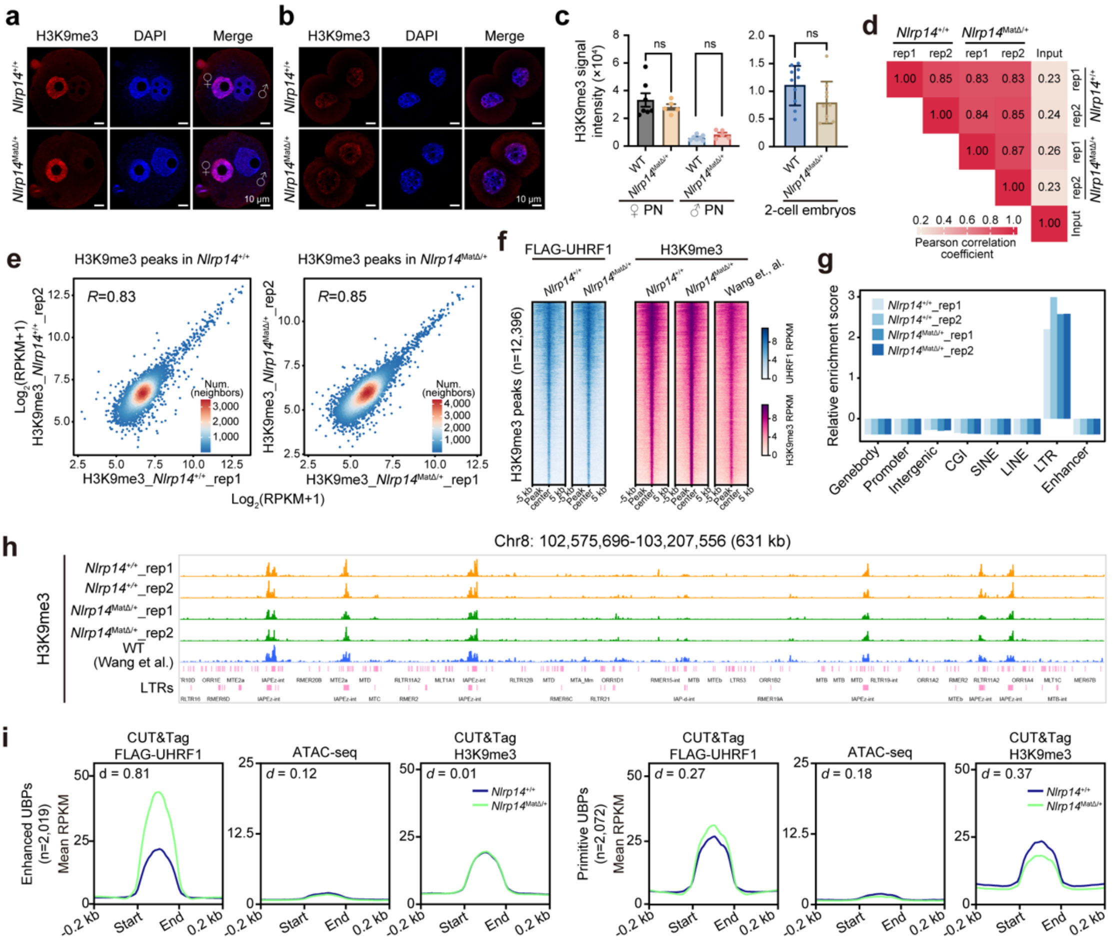

Supplementary Fig. S4 | H3K9me3 CUT&Tag of WT and *Nlrp14$^{mat\text{-}KO}$* 2-cell embryos. a-b, Immunofluorescence staining of H3K9me3 in *Nlrp14$^{mat\text{-}KO}$* zygotes (WT, n= 7; KO, n = 5) and 2-cell embryos (WT, n= 5; KO, n = 5). **c,** Quantification of H3K9me3 signal intensity in male and female pronuclei and 2-cell embryos from WT and *Nlrp14$^{mat\text{-}KO}$* embryos, normalized to DAPI. Statistical significance was assessed using a two-tailed Student's *t*-test. For zygote: WT, n = 7; KO, n = 5. For 2-cell: WT, n = 5; KO, n = 5. ns, not significant. **d,** Heatmap showing the Pearson correlation coefficient of H3K9me3 signals in mouse WT and *Nlrp14$^{mat\text{-}KO}$* 2-cell embryos. CUT&Tag using an anti-IgG antibody in WT 2-cell embryos served as a negative control (Input). **e,** Scatter plots showing the correlation of H3K9me3 CUT&Tag data between biological replicates of WT and *Nlrp14$^{Mat\text{-}KO}$* 2-cell embryos. *R* represents the Pearson correlation coefficient. Enrichment per 5-kb bin is shown in log_2(RPKM+1) values. **f,** Heatmap showing UHRF1 binding signals and H3K9me3 signals in approximately 5-kb regions of H3K9me3 peaks. H3K9me3 ChIP-seq data from Wang

et., al. was also included. **g,** Relative enrichment score of H3K9me3 peaks identified in WT and *Nlrp14*$^{Mat\text{-}KO}$ 2-cell embryos across each genomic element. **h,** Track plot showing H3K9me3 signals in WT and *Nlrp14*$^{Mat\text{-}KO}$ 2-cell embryos. H3K9me3 ChIP-seq data from Wang et., al. was also included to evaluate the data consistency. **i,** Profile plots of FLAG-UHRF1, ATAC-seq and H3K9me3 signals across enhanced and primitive UBPs in WT and *Nlrp14*$^{Mat\text{-}KO}$ 2-cell embryos. Effect sizes are quantified using Cohen’s d and indicated in the figure. Cohen’s d ⩾ 0.8 is considered a large effect and interpreted as biological meaningful, whereas smaller effect sizes are regarded as minor differences.

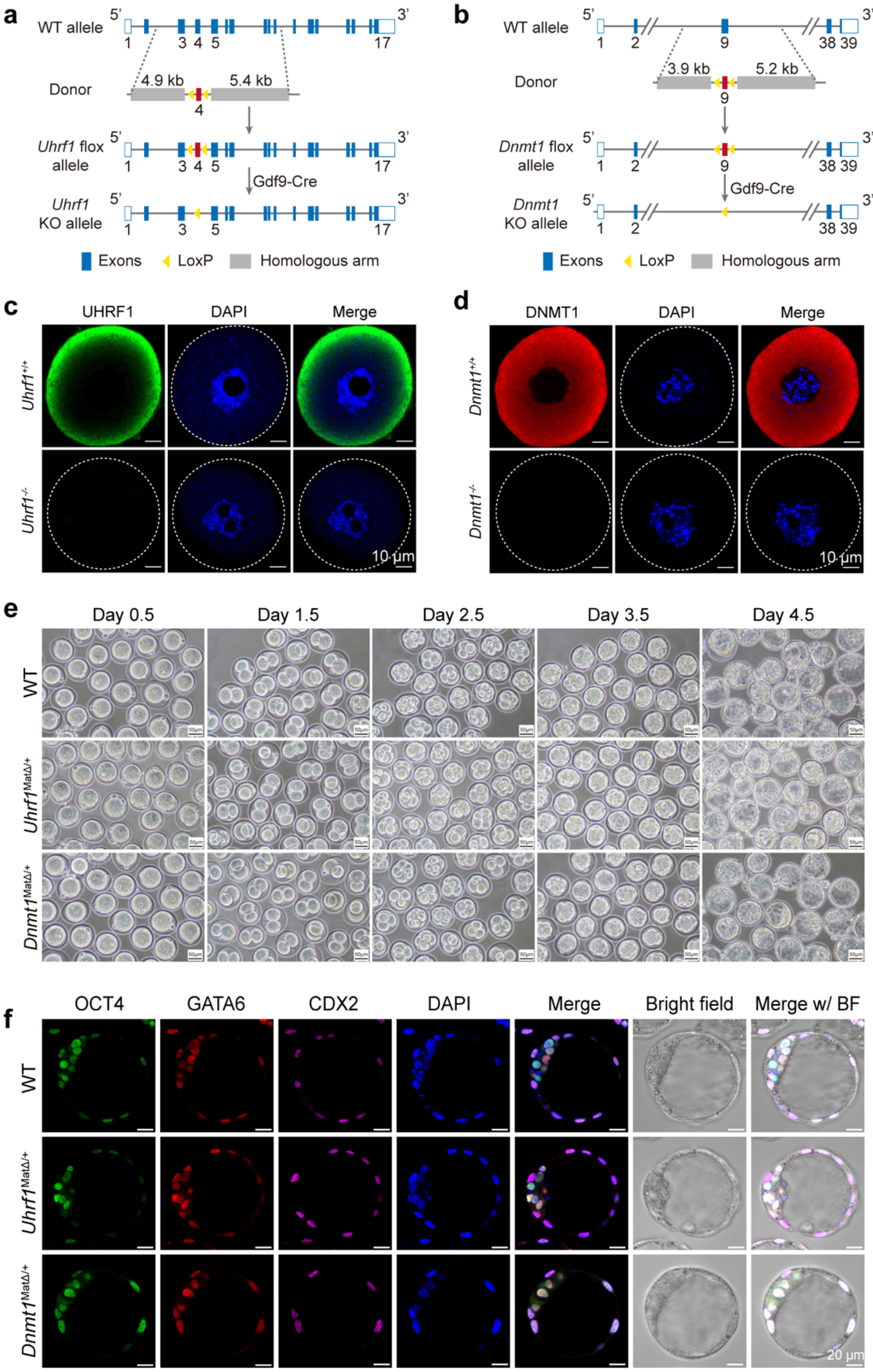

Supplementary Fig. S5 | Validation of *Uhrf1* and *Dnmt1* mat-CKO embryos. a-b, Strategies for generating *Uhrf1* conditional knockout (CKO) (**a**) and *Dnmt1* CKO mice (**b**). **c-d,** Immunofluorescence staining of UHRF1 and DNMT1 in WT, *Uhrf1* KO (**c**) or *Dnmt1* KO (**d**) GV oocytes. For UHRF1 staining: WT, n = 3; *Uhrf1* KO, n

= 4. For DNMT1 staining: WT, n = 4; *Dnmt1* KO, n = 6. **e,** Representative images of cleavage embryo morphology of WT, *Uhrf1*$^{mat\text{-}KO}$ and *Dnmt1*$^{mat\text{-}KO}$ embryos. **f,** Immunofluorescence staining of OCT4, GATA6, and CDX2 in WT (n = 19), *Uhrf1*$^{mat\text{-}KO}$ (n = 11) and *Dnmt1*$^{mat\text{-}KO}$ (n = 17) blastocysts.

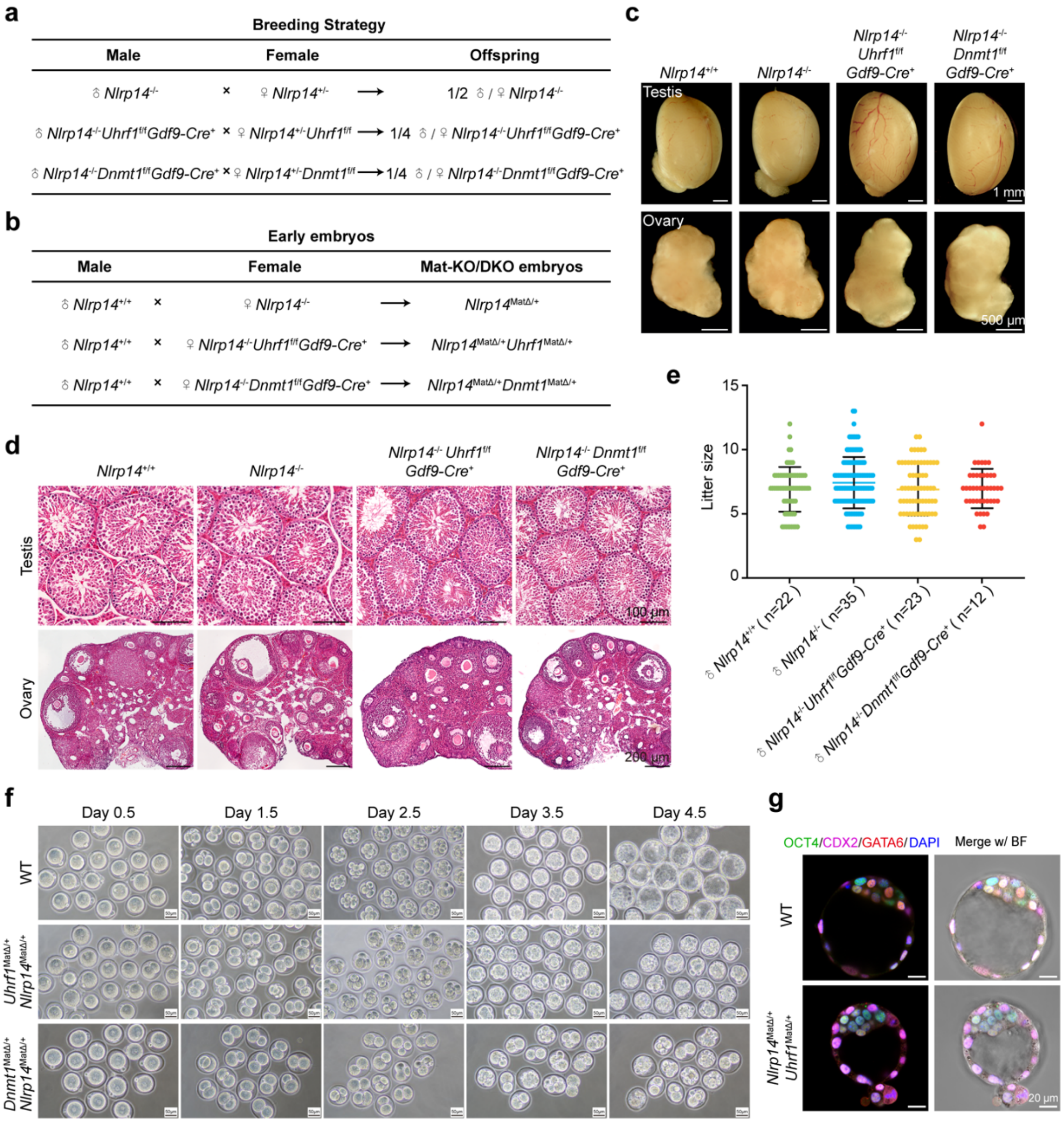

Supplementary Fig. S6 | The strategy and validation of *Uhrf1* & *Nlrp14* or *Dnmt1* & *Nlrp14* double CKO mice. a-b, Strategy for breeding and generating KO mice and early embryos. **c,** Representative images of testis and ovary morphology from WT (n = 6), *Nlrp14* KO (n = 3), [$Nlrp14^{-/-}$ $Uhrf1^{f/f}$ $Gdf9\text{-}Cre^{+}$] (n = 3) and [$Nlrp14^{-/-}$ $Dnmt1^{f/f}$ $Gdf9\text{-}Cre^{+}$] (n = 3) mice. **d,** Representative H&E staining images of testis and ovary morphology from WT (n = 6), *Nlrp14* KO (n = 3), [$Nlrp14^{-/-}$ $Uhrf1^{f/f}$ $Gdf9\text{-}Cre^{+}$] (n = 3) and [$Nlrp14^{-/-}$ $Dnmt1^{f/f}$ $Gdf9\text{-}Cre^{+}$] (n = 3) mice. **e,** Scatter plot showing the litter sizes of WT, *Nlrp14* KO, [$Nlrp14^{-/-}$ $Uhrf1^{f/f}$ $Gdf9\text{-}Cre^{+}$] and [$Nlrp14^{-/-}$ $Dnmt1^{f/f}$ $Gdf9\text{-}Cre^{+}$] male mice. The error bars represent the means ± SD. **f,** Representative images of cleavage embryo morphology from WT, $Uhrf1\&Nlrp14^{mat\text{-}DKO}$ and $Dnmt1\&Nlrp14^{mat\text{-}DKO}$ embryos. **g,** Immunofluorescence staining of OCT4, GATA6, and CDX2 in WT (n = 5) and $Uhrf1\&Nlrp14^{mat\text{-}DKO}$ (n = 4) blastocysts.

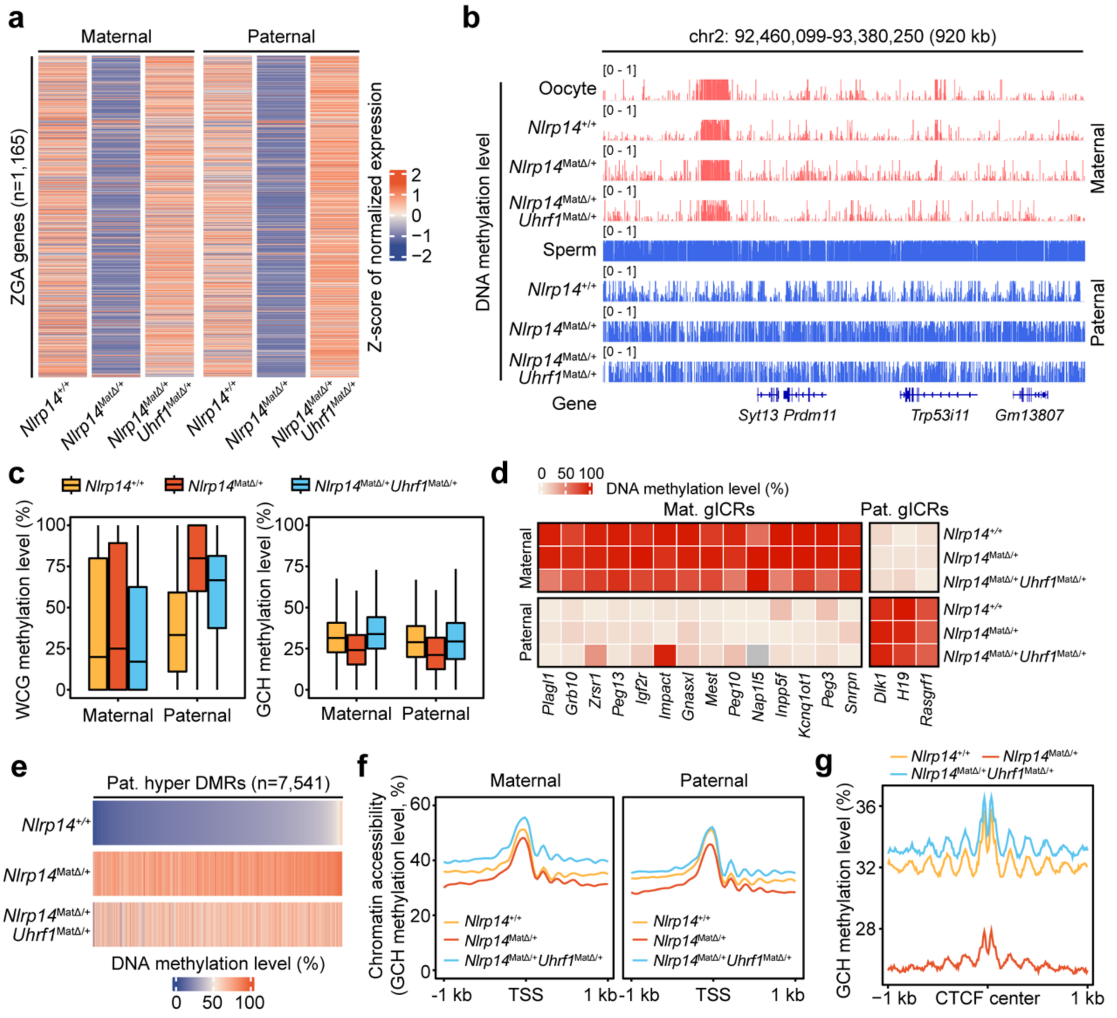

Supplementary Fig. S7 | The transcriptome, chromatin accessibility, and DNA methylome of *Uhrf1* & *Nlrp14* double KO embryos. a, Heatmap showing Z-scored normalized expression levels of ZGA genes in the maternal and paternal genomes of WT, *Nlrp14*$^{mat-KO}$ and *Uhrf1&Nlrp14*$^{mat-DKO}$ 2-cell embryos. **b,** Track plots showing representative parent-specific DNA methylation levels in MII oocytes, sperm, and WT, *Nlrp14*$^{mat-DKO}$, *Uhrf1&Nlrp14*$^{mat-DKO}$ 2-cell embryos. **c,** Box plots showing parental-specific WCG (left) and GCH (right) methylation levels in WT, *Nlrp14*$^{mat-KO}$, and *Uhrf1&Nlrp14*$^{mat-DKO}$ 2-cell embryos. Methylation levels were calculated on 1-kb tiles. **d,** Heatmap showing the DNA methylation levels of parental gICRs in WT, *Nlrp14*$^{mat-KO}$ and *Uhrf1&Nlrp14*$^{mat-DKO}$ 2-cell embryos. **e,** Heatmap showing the DNA methylation levels of paternal hyper DMRs (Yan *et al*., 2023) in WT, *Nlrp14*$^{mat-KO}$ and *Uhrf1&Nlrp14*$^{mat-DKO}$ 2-cell embryos. **f,** Line plots showing chromatin accessibility of TSS 1-kb flanking regions in WT, *Nlrp14*$^{mat-KO}$ and *Uhrf1&Nlrp14*$^{mat-DKO}$ 2-cell

embryos. **g,** Line plots showing GCH methylation levels at CTCF-binding motifs and 1-kb flanking regions in WT, *Nlrp14*$^{mat\text{-}KO}$ and *Uhrf1&Nlrp14*$^{mat\text{-}DKO}$ 2-cell embryos.

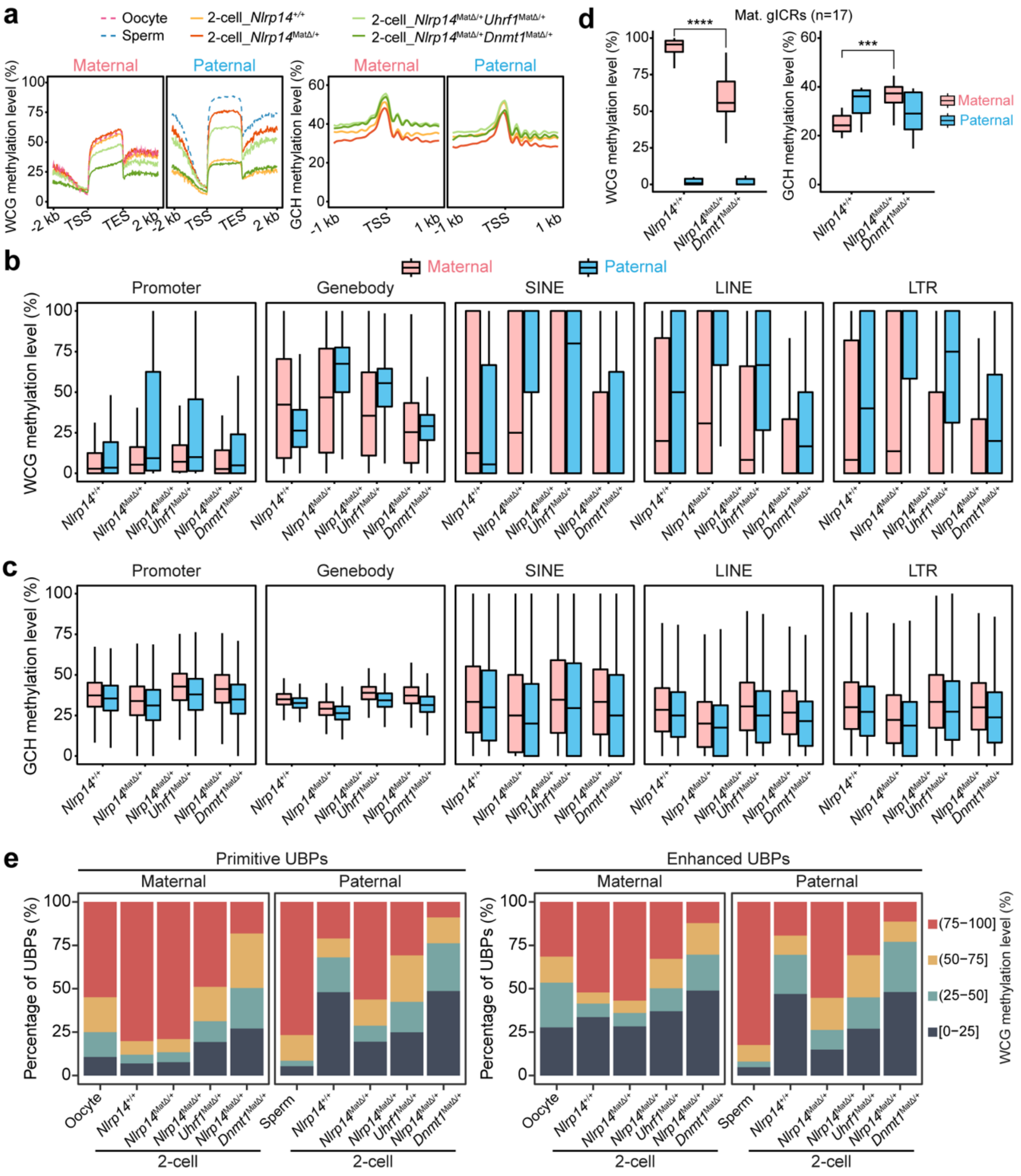

Supplementary Fig. S8 | Single-cell multi-omics analysis of *Dnmt1* & *Nlrp14* maternal double KO embryos. a, Line plots showing WCG methylation levels in the gene body (left) and GCH methylation levels in the TSS 1-kb flanking regions (right) in WT, *Nlrp14*$^{mat\text{-}KO}$, *Uhrf1&Nlrp14*$^{mat\text{-}DKO}$ and *Dnmt1&Nlrp14*$^{mat\text{-}DKO}$ 2-cell embryos. **b-c,** WCG (**c**) and GCH (**d**) methylation levels of representative genomic elements and repeats in WT, *Nlrp14*$^{mat\text{-}KO}$, *Uhrf1&Nlrp14*$^{mat\text{-}DKO}$ and *Dnmt1&Nlrp14*$^{mat\text{-}DKO}$ 2-cell embryos. **d,** Box plots showing WCG and GCH methylation levels of maternal gICRs in WT and *Dnmt1&Nlrp14*$^{mat\text{-}DKO}$ 2-cell embryos. Statistical significance was assessed using two-tailed Student's *t*-test. **e,** Stacked bar plots showing the

percentages of UBPs with categorical DNA methylation levels in MII oocytes, sperm, and WT, *Nlrp14*$^{\text{mat-KO}}$, *Nlrp14&Uhrf1*$^{\text{mat-DKO}}$, *Nlrp14&Dnmt1*$^{\text{mat-DKO}}$ 2-cell embryos. DNA methylation levels are color-coded.

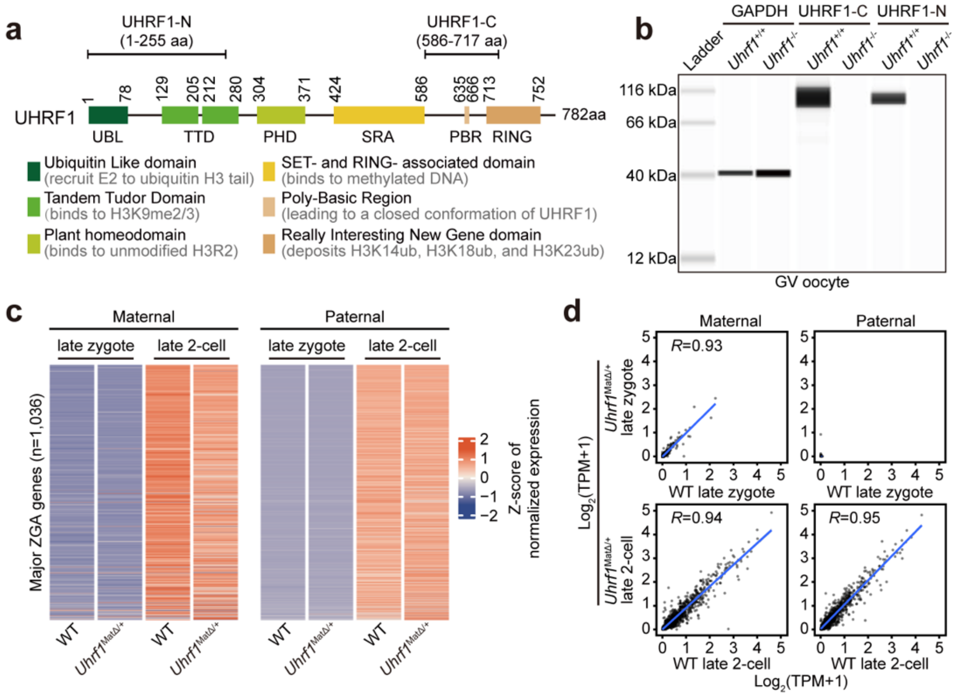

Supplementary Fig. S9 | Primitive UHRF1 binding in WT embryos was not associated with ZGA. a, Structure and function of the UHRF1 domains. **b,** WES analysis of UHRF1 in WT and *Uhrf1* KO GV oocytes using an UHRF1-N antibody (immunogenic region: aa 1-255 of UHRF1) and an UHRF1-C antibody (immunogenic region: aa 566-717 of UHRF1). GAPDH was used as a normalization control. **c,** Heatmap showing normalized expression of major ZGA genes in the parental genome in WT and *Uhrf1$^{mat\text{-}KO}$* zygotes and 2-cell embryos. **d,** Scatter plots showing the correlation of ZGA gene expression between WT and *Uhrf1$^{mat\text{-}KO}$* embryos in each parental genome. *R*, Pearson correlation coefficient.

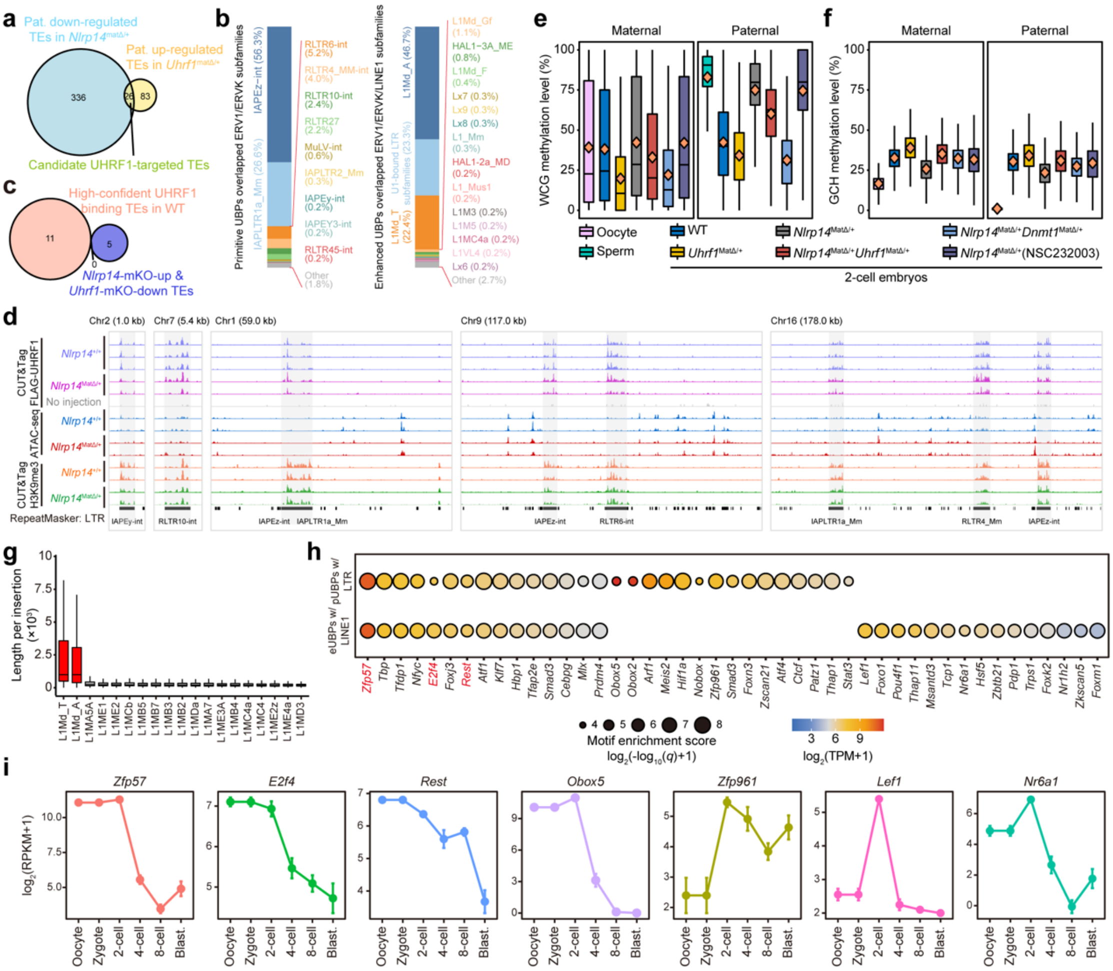

Supplementary Fig. S10 | Role of UHRF1 in regulating TEs activity across the entire genome at the onset of mammalian development. a, Overlap of paternal upregulated TEs in *Uhrf1$^{mat\text{-}KO}$* and downregulated TEs in *Nlrp14$^{mat\text{-}KO}$* 2-cell embryos when compared with WT 2-cell embryos. The differentially expressed TEs were defined in Fig. 5b. **b,** Stacked plots show the proportion of UBPs overlapping each subfamily. Primitive UBPs were intersected with insertion sites of ERV1 and ERVK subfamilies, as permutation analysis indicated significant enrichment of primitive UBPs in these TE classes but not in others. Similarly, enhanced UBPs were intersected with ERV1, ERVK, and LINE1 subfamilies based on permutation-defined enrichment. TE subfamilies with ⩾3 independent UBP overlaps were retained as high-confidence UHRF1 targets and are labeled with their names and overlap numbers. “U1-bound LTR” denotes LTR subfamilies identified as high-confidence targets based on primitive UBP overlap. Subfamilies with <3 overlaps are grouped as “Other”. **c,** Overlap of *Uhrf1$^{mat\text{-}KO}$*-down & *Nlrp14$^{mat\text{-}KO}$*-up TEs (identified in Fig. 5b)

and high-confident UHRF1 binding TEs in WT 2-cell embryos. **d,** Track plot showing the representative UHRF1 targeted LTR loci. The signals of chromatin accessibility (ATAC) and H3K9me3 in these regions in WT and *Nlrp14*$^{mat\text{-}KO}$ 2-cell embryos were also shown. The rectangular shadow box represents the UBP regions. Chromosomal coordinate information for the regions shown in this figure includes: Chr2: 157,475,036-157,484,987 (1.0 kb), Chr7: 69,278,848-69,284,244 (5.4 kb), Chr1: 16,710,633-16,769,745 (59.0 kb), Chr9: 6,748,010-6,865,455 (117.0 kb), Chr16: 76,025,650-76,204,383 (178.9 kb). **e-f,** Box plots showing the WCG methylation level (**e**) and GCH methylation level (**f**) on global 5-kb tiles in oocyte, sperm, and 2-cell embryos from WT and five mouse models generated in this study. **g,** Box plots showing the insertion length distribution of representative LINE1 subfamilies. **h,** Motif enrichment analysis of (top panel, pUBPs with LTR) primitive UBPs overlapping four LTR subfamilies (IAPLTR1a_Mm, RLTR27, IAPEz-int, and IAPEy-int), and (bottom panel, eUBPs with LINE1) enhanced UBPs overlapping two LINE1 subfamilies (L1Md_A and L1Md_T). Circle size indicates the degree of motif enrichment, and circle color represents the expression level of the corresponding transcription factor in mouse zygotes. **i,** Expression dynamics of representative transcription factors selected from the enriched motifs in panel (h) across developmental stages from oocyte to blastocyst.
